# Fully conjugated ladder polymers

**DOI:** 10.1039/c7sc00154a

**Published:** 2017-02-17

**Authors:** Jongbok Lee, Alexander J. Kalin, Tianyu Yuan, Mohammed Al-Hashimi, Lei Fang

**Affiliations:** a Department of Chemistry , Texas A&M University , 3255 TAMU , College Station , TX 77843 , USA . Email: fang@chem.tamu.edu; b Materials Science & Engineering Department , Texas A&M University , 3003 TAMU , College Station , TX 77843 , USA; c Department of Chemistry , Texas A&M University at Qatar , P.O. Box 23874 , Doha , Qatar

## Abstract

Fully conjugated ladder polymers (cLPs), in which all the backbone units on the polymer main-chain are π-conjugated and fused, have attracted great interest owing to their intriguing properties, remarkable chemical and thermal stability, and potential suitability as functional organic materials. The synthesis of cLPs can be, in general, achieved by two main strategies: single-step ladderization and post-polymerization ladderization. Although a variety of synthetic methods have been developed, the chemistry of cLPs must contend with structural defects and low solubility that prevents complete control over synthesis and structural characterization. Despite these challenges, cLPs have been used for a wide range of applications such as organic light emitting diodes (OLEDs) and organic field effect transistors (OFETs), paralleling developments in processing methods. In this perspective, we discuss the background of historical syntheses including the most recent synthetic approaches, challenges related to the synthesis and structural characterization of well-defined cLPs with minimum levels of structural defects, cLPs' unique properties, and wide range of applications. In addition, we propose outlooks to overcome the challenges limiting the synthesis, analysis, and processing of cLPs in order to fully unlock the potential of this intriguing class of organic materials.

## Abstract 2

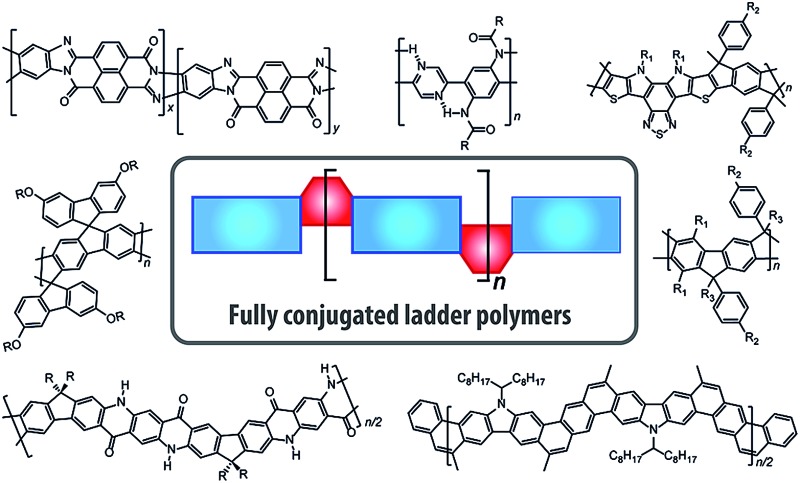
Syntheses, properties and applications of fully conjugated ladder polymers are reviewed, together with an outlook to future opportunities and challenges.

## 1. Introduction

Fully conjugated ladder polymers (cLPs) are an intriguing subset of macromolecules. Their development has relied on a wide scope of synthetic strategies to obtain a host of unique structures and materials useful for their physical, optical, and chemical properties.^1–3^ In general, ladder polymers are multiple stranded polymers with periodic linkages connecting the strands, resembling the rails and rungs of a ladder, and giving an uninterrupted sequence of adjacent rings that share two or more atoms.^4^ Conjugated ladder polymers (cLPs) are a specific subtype of ladder polymers in which all the fused rings in the backbone are π-conjugated. In addition, they are distinct from conventional conjugated polymers in that the fused-ring constitution restricts the free torsional motion in between the aromatic units along the backbone.

**Fig. 1:**
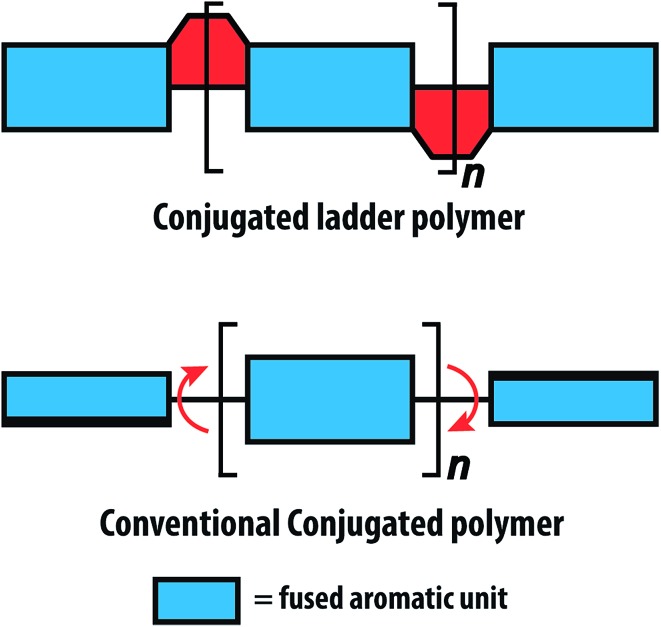
Graphical representation of conjugated ladder polymer (cLP) and conventional conjugated polymer with free torsional motions.

Stemming from the fused backbone, cLPs exhibit extraordinary thermal, chemical, and mechanical stability.^3,5–7^ Because of the diminished torsional defects, cLPs with fully coplanar backbones promise coherent π-conjugation,^8^ fast intra-chain charge transport,^9^ long exciton diffusion length,^10^ and strong π–π stacking interactions.^1^ In contrast, the aromatic repeating units of conventional conjugated polymers tend to adopt non-zero dihedral angles either because of torsional strain or thermal fluctuation (Fig. 1). Such torsional defects partially break the conjugation along the polymer backbone, resulting in decreased electronic delocalization, widened band gaps, increased numbers of trapped charges, and less effective intermolecular coupling.^8,11^

Since the synthesis of poly(benzimidazole benzophenanthroline) (BBL) **1** in the 1960s,^12^ many different cLPs have been prepared and investigated for various applications.^2,3^ Despite numerous reported syntheses of cLPs, the field has seen inherent synthetic challenges limiting the scope of usable precursors and reaction designs. Limitations are mainly related to several issues; (i) there are relatively few synthetic strategies available to efficiently construct defect-free structures; and (ii) poorly soluble products caused by backbone rigidity and coplanarity. In order to construct a well-defined cLP, the conversion of the ring annulation reaction must be nearly quantitative without undesired side reactions such as intermolecular cross-linking. Due to the lack of backbone rotation, at least one of the building blocks' reaction sites should possess *C*_2h_ symmetry in order for the rigid backbone to extend linearly. Furthermore, adequate solubilizing groups on the backbone of the cLPs are required to allow the reaction to complete while still obtaining soluble products for the subsequent characterization and processing.

From initial synthesis, through to characterization, analysis, and finally as potential end-user applications, cLPs face a number of unique challenges not found in other organic materials, originating from their rigid ribbon-like structures.^3,13^ The low solubility and backbone rigidity of the polymers limit the effectiveness of common polymer analysis techniques such as structural elucidation by NMR or molecular weight estimation by size exclusion chromatography (SEC). The low solubility also impedes simple solution processing methods in some cases. In this context, unique methods have been developed to circumvent these barriers, leading to more straightforward syntheses and widespread uses of cLPs.

Despite the aforementioned challenges in cLPs, their exceptional stability and promising electronic properties have prompted exploration in various optical and electronic applications, such as OLEDs^2,5,14–16^ and OFETs,^17–21^ among others. In a large number of examples, cLP optoelectronic properties surpassed that of their non-ladder type counterparts.

In this perspective, our focus will center on the general synthetic strategies and specific examples of cLPs followed by discussion of chemical and engineering challenges associated with these materials. The demonstrated functions and potential applications of cLPs on multiple fronts are also discussed and outlined. Please note that conjugated step-ladder polymers^3^ (conjugated polymers composed of oligomeric ladder-type building blocks connected by single-stranded σ bonds) are not discussed in this perspective. Fused-ring π-conjugated oligomers are also excluded from this perspective due to the limitation on the page and reference number.

## 2. Review/discussion

### 2-1. General synthetic strategies

**Fig. 2:**
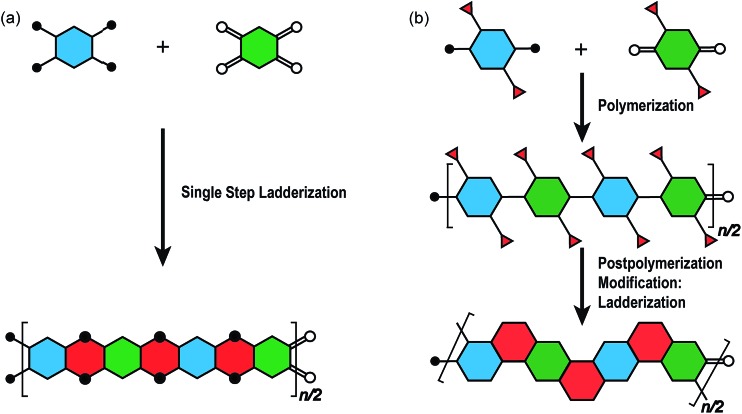
Graphical synthetic approaches to construct a ladder polymer. (a) Single-step ladderization and (b) post-polymerization modification: ladderization.

The synthesis of a well-defined cLP must fulfill several criteria; (i) reasonable solubility and (ii) quantitative conversion in the ring-closing reactions. In addition, the issues impacting the degree of polymerization and polydispersity must also be taken into consideration. Therefore, the development of an efficient and versatile synthetic strategy is indispensable to explore the potential for a functional cLP. In general, two distinct approaches can be employed to construct a fully conjugated ladder-type structure (Fig. 2). One is single-step “ladderization” that constructs two strands of bonds simultaneously, such as polycondensation of tetra-functional monomers or repetitive Diels–Alder cycloaddition. The other approach relies on post-polymerization annulation. In this two-step approach, a pre-functionalized single-stranded conjugated polymer is first prepared, followed by the ladderization steps in which the functional groups cyclize to form the second strand of bonds. This stepwise approach provides a wider scope of applicable synthetic methods and monomeric building blocks. For this strategy, however, it is essential to ensure high conversion of the post-polymerization annulation reaction, while keeping good solubility of the reaction intermediate to achieve a well-defined ladder polymer with minimum levels of structural defects. Herein, we introduce the backgrounds and features of important cLP syntheses, discuss developments in the last decade, and offer synthetic perspective on cLPs.

**Fig. 3:**
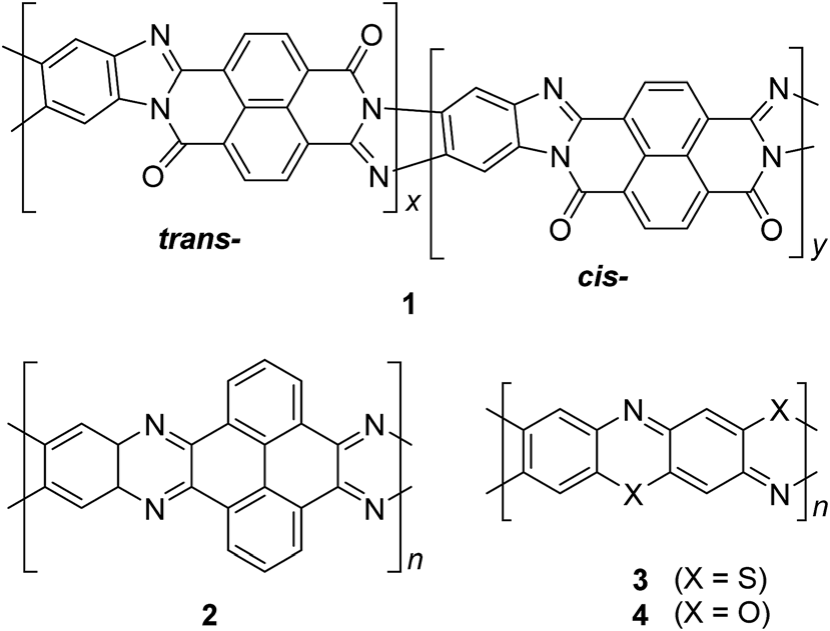
Chemical structures of *trans* and *cis* poly(benzimidazole benzophenanthroline) (BBL) **1**, polyquinoxaline (PQL) **2**, poly(phenthiazine) (PTL) **3**, and poly(phenooxazine) (POL) **4**.

### 2-2. Single-step ladderization

In the late 1960s, Van Deusen reported the synthesis of BBL **1** for the purpose of thermally stable organic materials (Fig. 3).^12^ BBL **1** now represents one of the most extensively studied cLPs to date. The synthesis of BBL **1** was achieved by a single-step polycondensation of two tetrafunctional monomers (1,2,4,5-tetraaminobenzene and 1,4,5,8-tetracarboxynaphthalene) in polyphosphoric acid (PPA) solution. It can be viewed as a statistical copolymer of *cis* and *trans* isomeric repeating units. In parallel, one of these tetrafunctional monomers, 1,2,4,5-tetraaminobenzene, was also used in the construction of other cLPs with different comonomers by Stille *et al.* ^22,23^ Their first trial to prepare a polyquinoxaline (PQL) ladder polymer **2** with hydroxylketone and tetraamine monomers provided incomplete ladder formation resulting in low thermal stability due to the low reactivity of the hydroxyl group.^22^ By replacing the hydroxyketone monomer with a tetraketone monomer, *e.g.* 1,2,6,7-tetraketopyrene, where tautomerization is restricted, thermally stable PQL **2** was afforded in hexamethylphosphoramide (HMPA) solution at 180 °C.^23^ It is noteworthy to mention that the key monomer (tetraaminobenzene) for the synthesis of BBL **1** and PQL **2**, is not stable to air oxidation. Therefore, oxidative side-reactions may cause structural defects of the cLP product if the reaction was not handled in a rigorously oxygen free condition. Similar synthetic strategy was employed in exploring the synthesis of poly(phenthiazine) (PTL) **3** and poly(phenooxazine) (POL) **4** by Kim in the 1980s.^24^ These single-step polycondensed cLPs, however, can usually only be suspended in strong acids such as PPA or sulfuric acid, and are insoluble in common organic solvents. As a result, common solution phase characterization techniques (NMR and SEC) were not feasible to fully elucidate these structures.

**Scheme 1:**
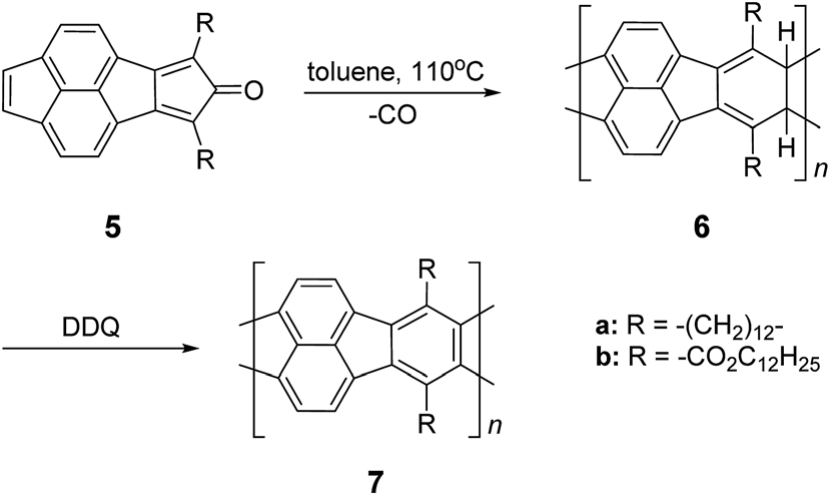
Synthesis of ladder polymer **7** by Diels–Alder reaction followed by dehydrogenation.

Nonetheless, a promising synthetic approach was reported by Schlüter and coworkers in the mid 1990s.^25,26^ The fully unsaturated ladder polymer backbone was achieved by using Diels–Alder reaction followed by dehydrogenation (Scheme 1). These two reactions both gave high conversions on small molecule model compounds. cLP **7a** was synthesized through an AB + AB step growth polymerization using one single monomer containing both diene and dienophile functionalities. The product was analyzed by elemental analysis, UV-vis spectroscopy, and cross polarization magic-angle spinning (CP-MAS) ^13^C NMR spectroscopy.^25^ The carbon resonance peak corresponding to saturated carbons in the backbone of **7a** disappeared after dehydrogenation. Compound **7a** was insoluble in common organic solvents even with a long looped alkyl solubilizing group. Furthermore, film formation was not possible even when using the low molecular weight fraction (*M*_n_ = 2–7 kg mol^–1^ by SEC). A different solubilizing group was also installed to improve the molecular weight of intermediate **6**.^26^ When an ester-linked dodecyl alkyl chain was used as the solubilizing group and the reaction was performed in an airtight ampoule, *M*_n_ of **6b** was relatively improved (34 kg mol^–1^ by SEC and 85 kg mol^–1^ by osmometry). After dehydrogenation, CP-MAS ^13^C NMR spectrum of product **7b** showed complete disappearance of the sp^3^ carbon on the polymer backbone. Product **7b**, however, was still insoluble in several organic solvents, preventing its analysis by solution-phase ^1^H NMR spectroscopy. Although single-step ladderization has been investigated for over 50 years, the methods have not been widely adopted as a general approach for cLP synthesis due to the limited availability of ideal multifunctional monomers and their related solubility issues.

**Scheme 2:**
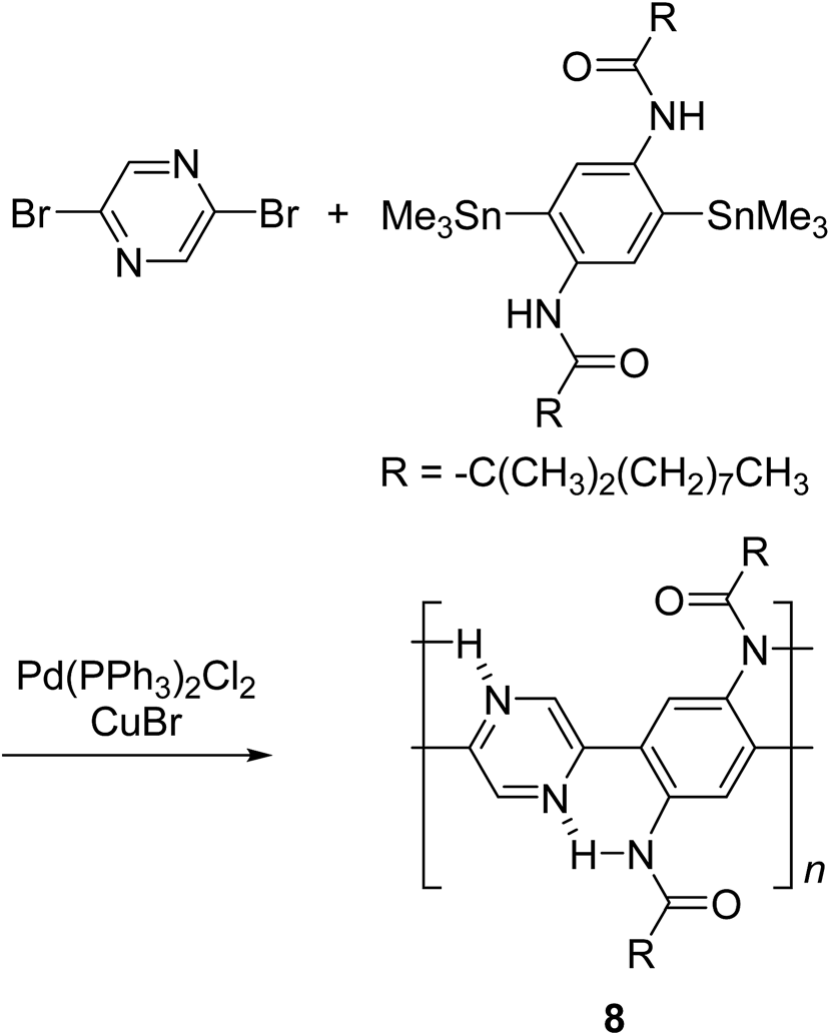
Synthesis of ladder polymer **8** by self-assembled intramolecular N–H interaction.

By using intramolecular non-covalent bonds, ladder-like conjugated backbones can be constructed through the formation of only one strand of covalent bonds. This strategy could also be considered as an interesting one-step approach to cLP-mimicking polymers. Through this approach, one strand of covalent bonds is formed through polymerization while another strand of non-covalent bonds can be generated simultaneously because of the dynamic and spontaneous nature of the non-covalent bonds. This approach was demonstrated in 1996, when Meijer and coworkers synthesized^27^ a ladder-like polymer using intramolecular hydrogen bonding between the nitrogen on 2,5-dibromopyrazine and an adjacently attached amide functionality (Scheme 2). In this case, although the intramolecular hydrogen bonding feature was observed by ^1^H NMR and IR spectroscopy, the synthesized polymer **8** was not able to adopt a fully coplanar structure along the backbone, due to the 2,2′ H–H steric repulsion on the neighboring benzene and pyrazine units.^28^ It is imperative that no steric effect should be present between neighboring rings in order to approach backbone coplanarity by using non-covalent interactions. Intramolecular dynamic bond-assisted coplanarization has been also reported using various kinds of non-covalent/coordination interactions such as N–H, S–N, and B–N interactions.^29–33^ In principle, these dynamic yet simultaneous bonding could be used in the future for a one step construction of cLPs. In general, pre-organized non-covalent interactions could provide an alternative method to construct a coplanar ladder-like polymer without the concerns of the ladderization efficiency or intermolecular cross-linking during a ladderization step. Furthermore, the dynamic nature of intramolecular bonding could allow a simple approach to actively control the torsional conformation and intermolecular packing while processing these polymers into the solid-state.

### 2-3. Two step approach: polymerization followed by ladderization

#### 2-3-1. Kinetic ring annulation

**Scheme 3:**
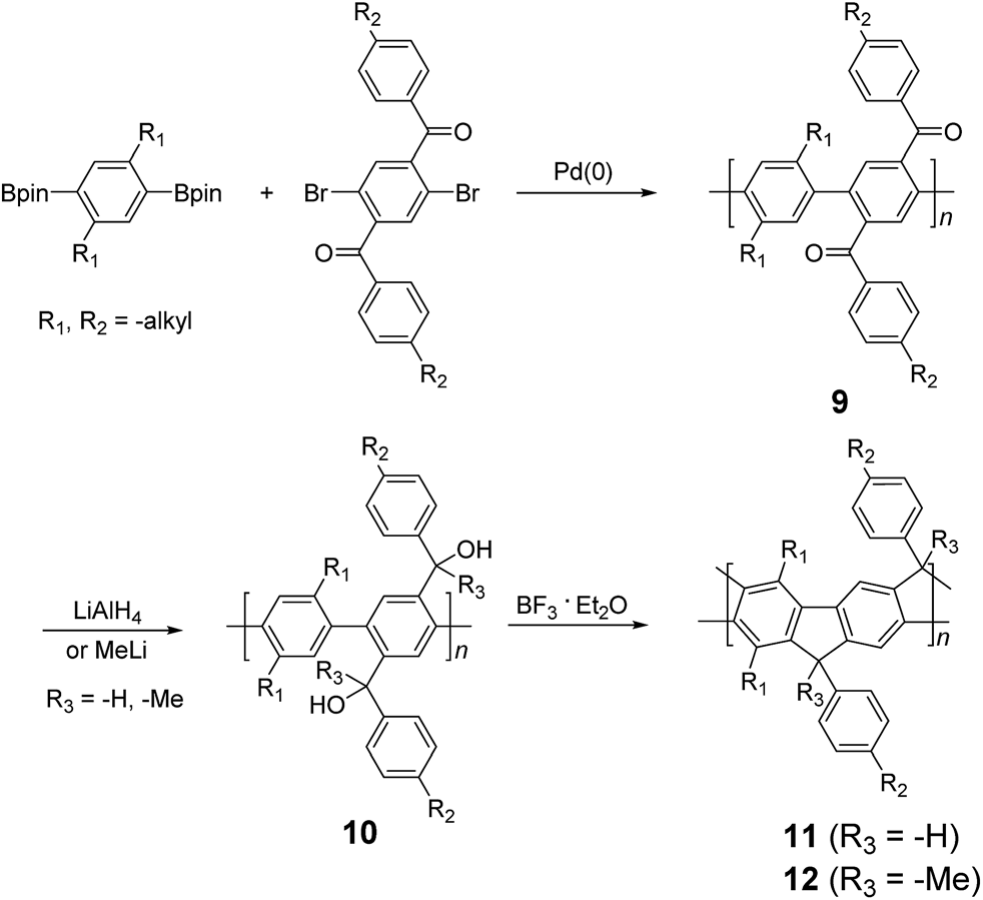
Synthesis of poly(*p*-phenylene) ladder polymers (LPPPs) **11** and **12** by Friedel–Craft ring annulation.

A widely used, Friedel–Crafts method to construct ladder-type poly(*para*-phenylene) structures was reported by Müllen and coworker in 1991.^34^ The fused-ring backbones are achieved by transition metal-mediated polymerization followed by electrophilic cyclization. The synthesis of ladder-type poly(*para*-phenylene)s (LPPPs) started with Suzuki polymerization of benzenebisboronic ester and aromatic ketone-functionalized dibromobenzene (Scheme 3). The ketone functional groups on the single-stranded intermediate **9** were reduced by lithium aluminum hydride or alkyllithium reagents. Eventually, Lewis acid-mediated Friedel–Crafts ring annulation afforded the double-stranded LPPP products. Due to the rigid coplanar backbone of LPPPs, their UV-vis spectra possess a well-resolved vibronic progression with a very small Stokes shift (4 nm).^35,36^ In addition, MeLPPP (R^3^ = Me) **12** showed identical photoluminescence (PL) spectra in solution and the thin film state, indicating that the molecular conformation does not change from the solvated state into the solid state.^2^ The key factor in this synthetic strategy is the steric hindrance on the bridgehead. It is essential that substituents on –CR^2^R^3^OH possess moderate steric hindrance (*i.e.*, R^3^ = H or alkyl).^2^ Less hindered substituents can lead to intermolecular cross-linking during the reaction, resulting in insoluble by-products. On the other hand, a bulkier substituent could prevent the ring annulation from completing. Later on, this synthetic strategy was expanded to prepare a number of different types of ladder polymers.^37–41^

**Scheme 4:**
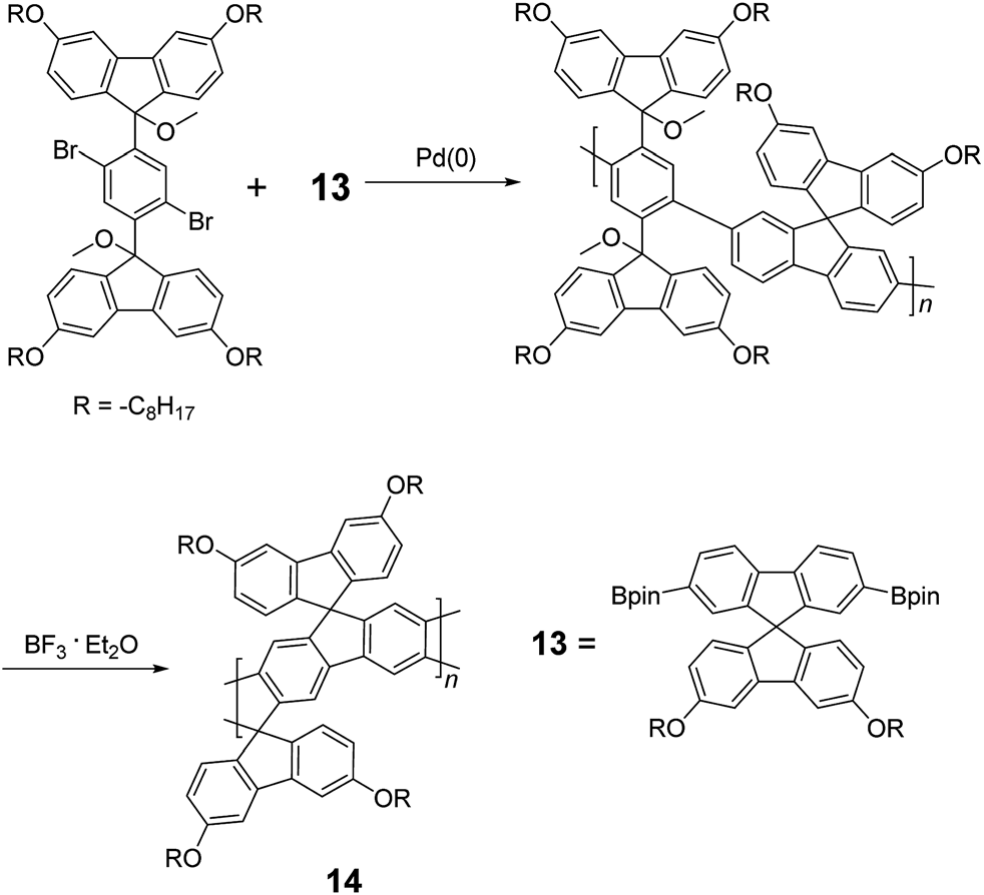
Synthesis of spiro-bridged LPPP **14** by Friedel–Craft ring annulation.

**Scheme 5:**
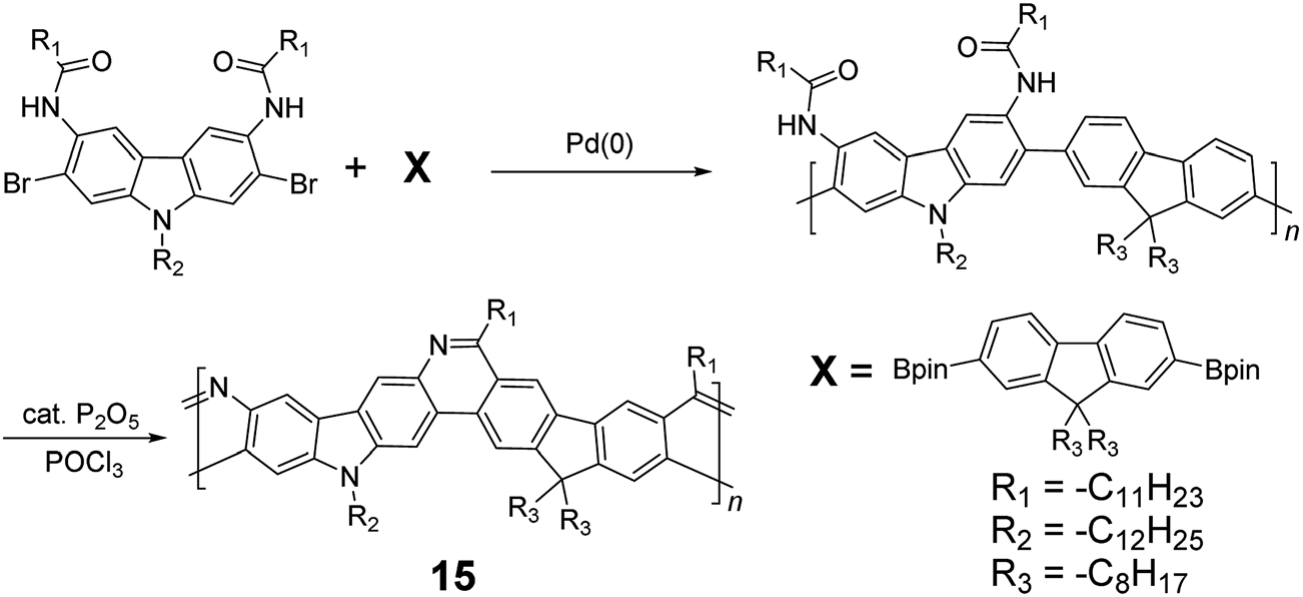
Synthesis of carbazole-fluorene-based ladder polymer **15** by Bischler-Napieralski cyclization.

**Scheme 6:**
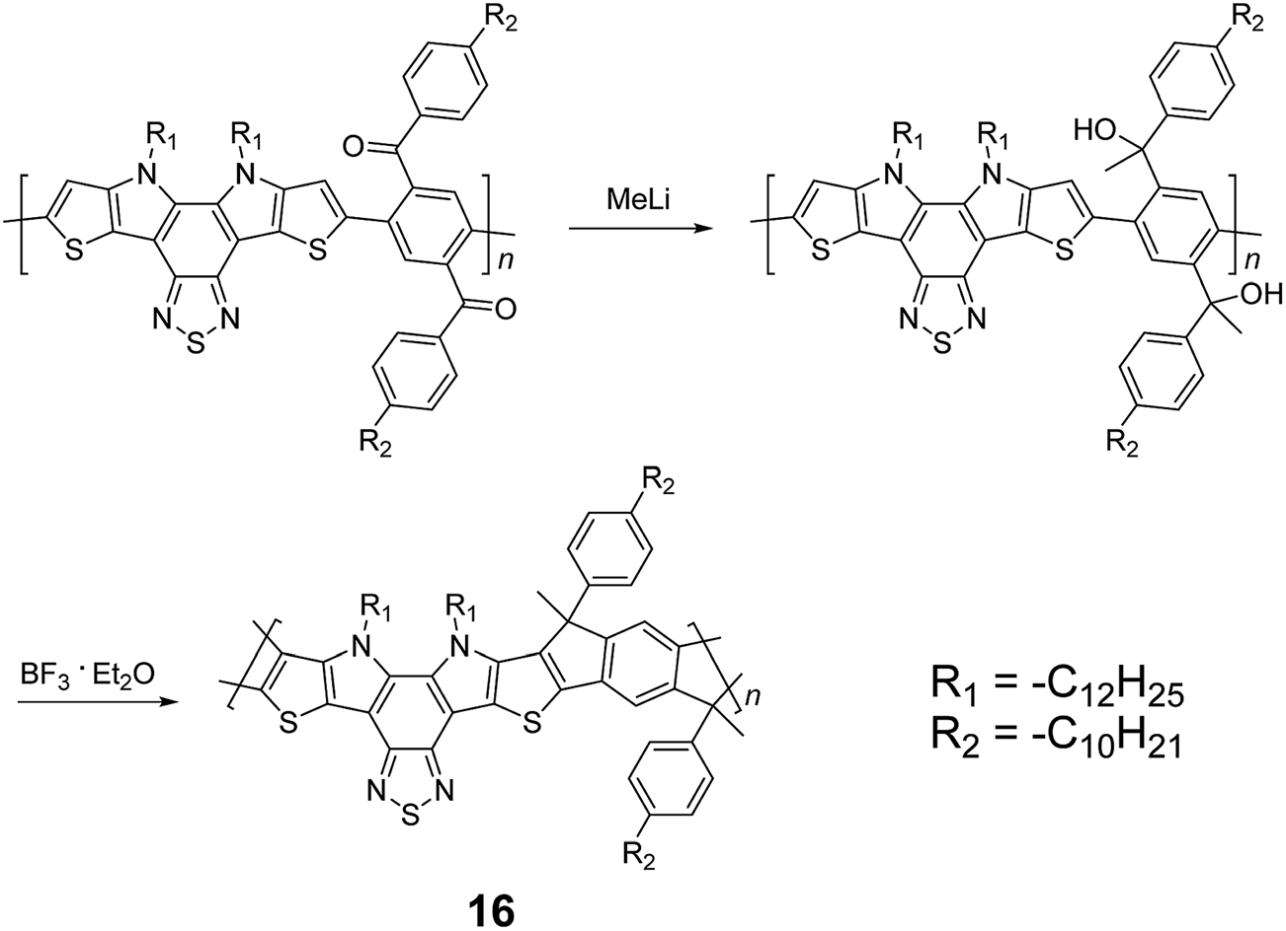
Synthesis of D–A type ladder polymer **16** by Friedel–Craft ring annulation.

Although ^1^H NMR and FT-IR spectroscopy detected no defect in LPPP polymers, careful investigations on the structure–property relationship of these polymers were performed to reveal small amounts of structural defects on their backbones which can be detrimental to the properties of the desired cLP materials.^42^ These defects were mainly caused by incomplete reduction of ketones that afforded monoalkylated fluorene backbones.^43,44^ As a result, these defect sites can be subsequently oxidized into fluorenones. The synthetic method was modified by Ma and coworkers to give a lower level of structural defects and thus better thermal stability.^45^ This method was further improved by Bo and coworkers,^5^ who introduced methoxy functional groups, replacing the hydroxyl groups to avoid keto defects (Scheme 4). In this case, a spiro-bridged solubilizing group was installed to minimize aggregation between the polymer chains. The bromide end groups of the conjugated polymer were end-capped using the monoboronic ester of **13** to afford a well-defined ladder-type structure. As a result, the synthesized spiro-bridged LPPP **14** showed typical properties of a rigid cLP – no obvious chromatic shift in the UV-vis and PL spectra between solution and the solid state was seen. In addition, the polymer exhibited a small Stokes shift of 2 nm, and excellent thermal and optical stability. The remarkable thermal stability was also demonstrated by the unchanged PL spectrum after annealing at 110 °C for 24 h in air. Bo and coworkers also reported the synthesis of a soluble imine-bridged ladder polymer **15** by Bischler-Napieralski cyclization (Scheme 5).^46^ The carbazole-fluorene conjugated polymer with dodecanamides was cyclized by POCl_3_ in the presence of P_2_O_5_ to form the imine bridge. It is interesting to note that the repeating units of the synthesized ladder polymer **15** lack a *C*_2h_ symmetry, resulting in a backbone which possesses an angular structure and does not extend in a straight manner. More recently, Scherf and coworkers reported a donor–acceptor (D–A) alternating ladder polymer **16**, fusing electron rich thiophene units and electron deficient benzothiadiazole (BTD) units in the backbone. The synthesis was achieved by reduction of the ketones followed by the ring-closing reaction in the presence of boron trifluoride (Scheme 6).^36^ The key to this successful synthetic design was the ability to pre-fuse the electron deficient BTD unit with thiophene, which avoided electrophilic cyclization on an already electron deficient aromatic unit.

**Scheme 7:**
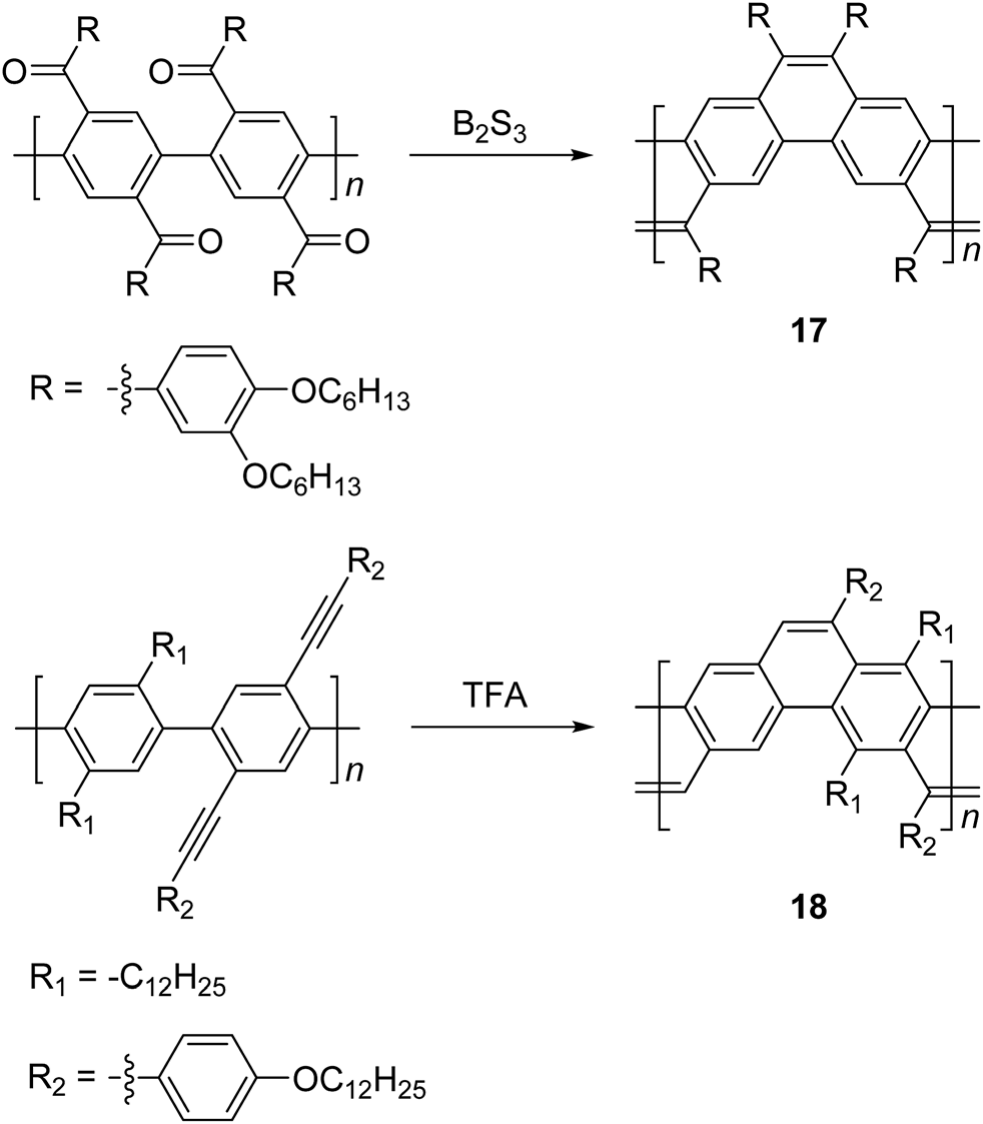
Synthesis of poly(*p*-phenacene)s **17** and **18** by carbonyl olefination and electrophile-induced cyclization, respectively.

Scherf and coworkers also reported^47^ the synthesis of ladder type poly(*p*-phenacene) derivatives by using Yamamoto coupling of a diketo-functionalized monomer, followed by carbonyl olefination in the presence of B_2_S_3_. Alternatively, the ladderization step could be also carried out by the McMurry reaction. In this report, polymer **17** with a linear side chain (4-decyloxyphenyl) resulted in a polymer which was marginally soluble, so that the product was soluble only at a low molecular weight (*ca.* 4 kg mol^–1^). The solubility and molecular weight of ladder type poly(*p*-phenacene) derivative **17** was improved by replacing the linear side chain with bulkier (3,4-dihexyloxy)phenyl units.^48^ A similar ladder-type backbone was also prepared by Swager and coworkers using electrophile-induced cyclization (Scheme 7).^49^ The acetylenic functional group on the conjugated polymer was cyclized in the presence of trifluoroacetic acid (TFA) to form aromatic rings to afford the ladder polymer **18**.

**Scheme 8:**
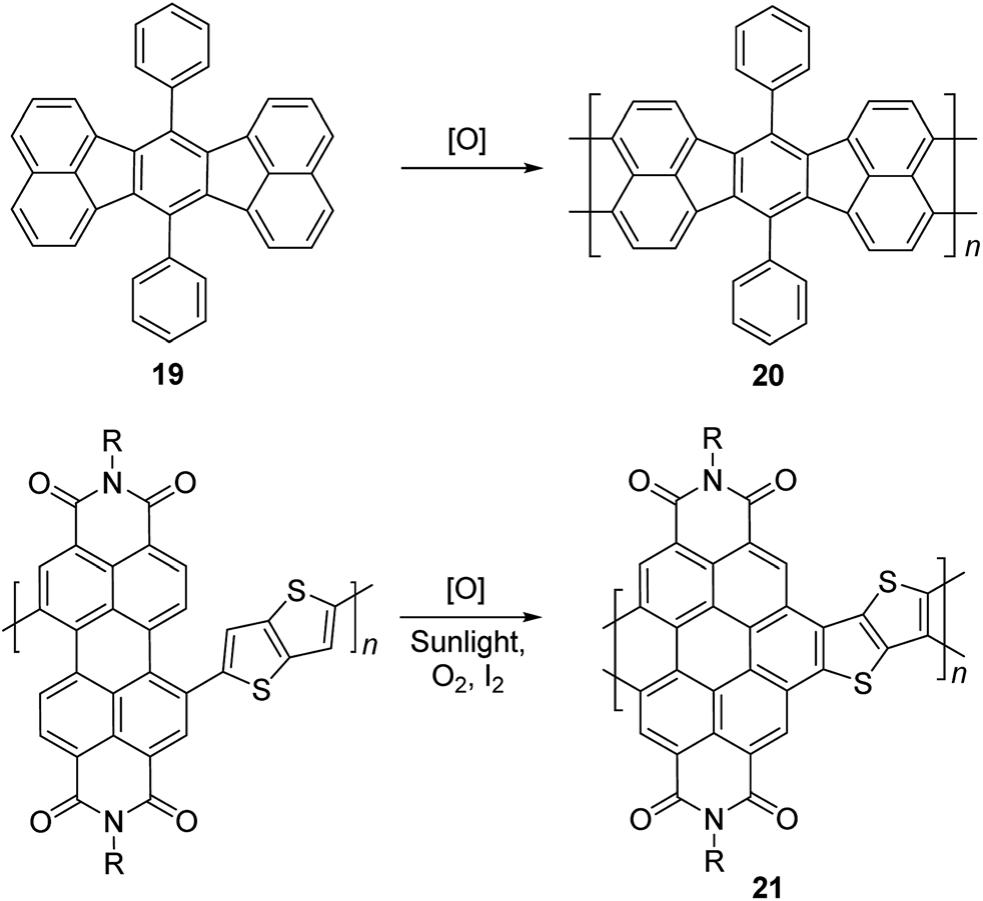
Synthesis of ladder polymer **20** by electrochemical oxidation and D–A type ladder polymer **21** by photochemical oxidation.

Electrochemical and photochemical oxidation reactions could also be employed to prepare cLPs (Scheme 8). Bard and coworkers described an electrochemical oxidation polymerization of precursor **19** to afford the postulated ladder polymer **20** deposited on the electrode surface. Because of the uncertain regioselectivity of the oxidative coupling process and the insolubility of **20**, the precise structure cannot be characterized by solution-phase analysis.^50^ Xiong and coworkers reported photocyclization of a conjugated polymer precursor under sunlight,^35^ to afford D–A ladder polymer **21** with a highly rigid and coplanar aromatic core. Due to the electron deficient nature of its perylene diimide components, **21** exhibited a low LUMO energy level of –3.98 eV, promising good n-type semiconducting behavior. Although the ladder polymer **21** had moderate solubility in common organic solvents at room temperature, structural characterization of the ladder backbone proved to be difficult.

**Scheme 9:**
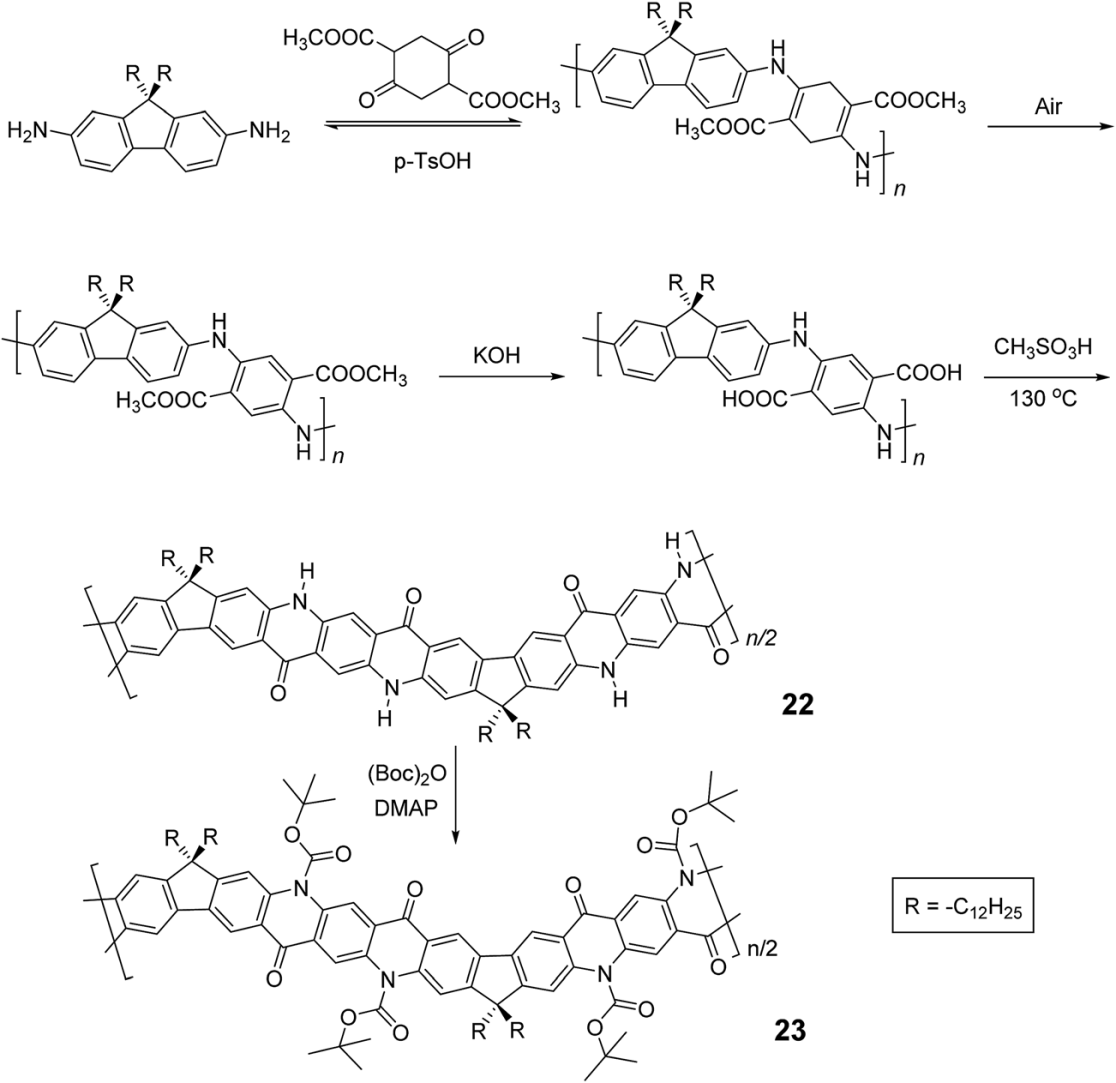
Metal catalyst-free synthesis of quinacridone derived ladder polymers **22** and **23**.

In the cases discussed above, metal catalyzed cross-coupling reactions were employed prevalently to construct the conjugated polymer precursors. A synthetic method free of precious metal catalyst, however, would suit better for scalable production of cLPs. Recently, Fang and coworkers reported a 3-step, metal-free synthesis of conjugated ladder polymer **22** derived from quinacridone (Scheme 9).^51^ Relying on imine polycondensation and a subsequent *in situ* oxidation in air, a conjugate backbone was constructed. The ring annulation was achieved through a kinetic process mediated by methanesulfonic acid. Although **22** was not soluble in common organic solvent, the structural elucidation was achieved indirectly by characterizing its soluble derivative **23**, which is functionalized with *t*-butoxycarbonyl (Boc) groups.

#### 2-3-2. Thermodynamic ring annulation

Distinct from kinetically controlled annulations, which require a careful selection of reagents and conditions for the efficient production of a single product due to the irreversibility of the reactions, thermodynamically controlled annulations, in principle, allow “error-checking” and “proof-reading” to push the reversible equilibrium to the most stable state.^52^ Because the goal in the synthesis of cLPs is usually to construct stable aromatic rings, thermodynamically controlled reactions should afford the desired product with higher yield and fewer structural defects such as unreacted sites or inter-chain crosslinking.

**Scheme 10:**
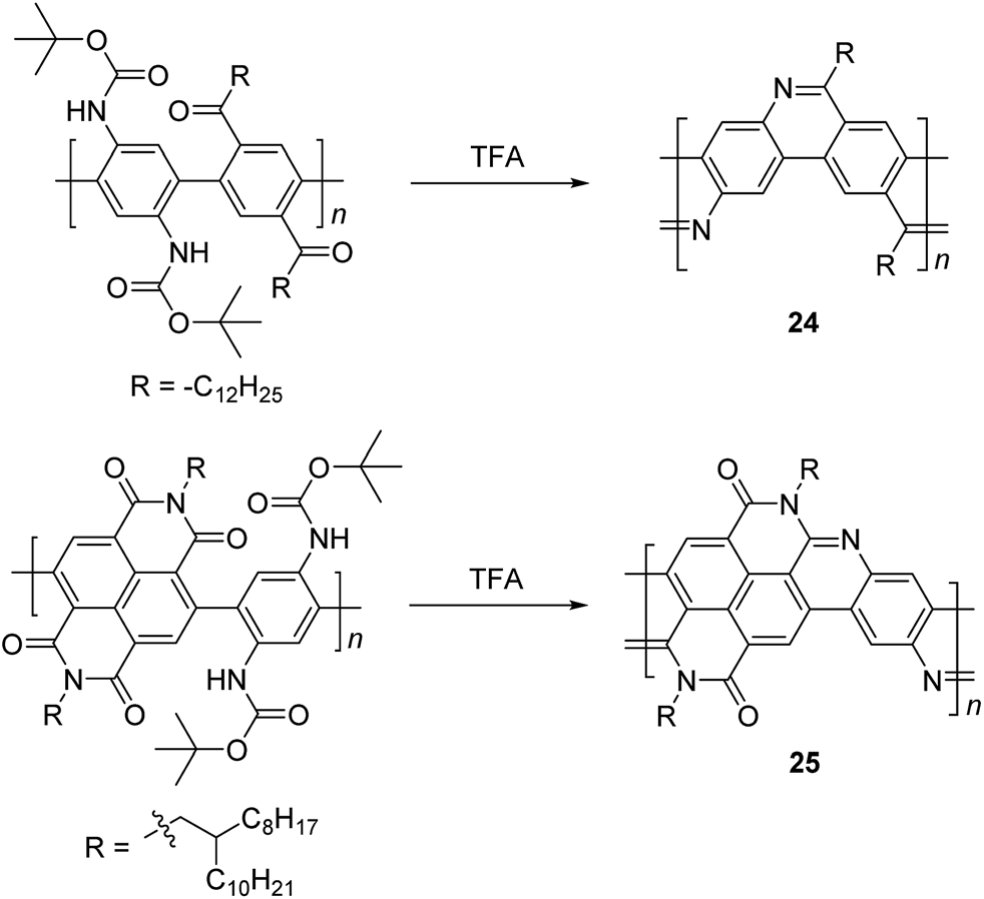
Synthesis of imine-bridged LPPP **24** and D–A type ladder polymer **25** by thermodynamically controlled imine condensation.

In the 1990s, Tour *et al.* reported a thermodynamically controlled reaction for post-polymerization modification that afforded cLP **24**.^53^ Imine-bridged LPPP **24** was synthesized by imine condensation of a conjugated polymer precursor (Scheme 10). To avoid unwanted imine condensation between free amines and ketones during the polymerization step, the amino group was protected by Boc group before the polymerization. In several small molecule model reactions, this method afforded fused aromatic ring formation and showed nearly quantitative conversions. The conjugated polymer was converted into imine-bridged cLP **24** by deprotection of the Boc group in the presence of TFA. However, **24** was only soluble in TFA, which can result in the protonation of the nitrogen atoms along the polymer chains and could partially dissociate the C=N bond; therefore, the structural analysis of unmodified **25** remained unclear. Luscombe and coworkers also reported solution processable imine-bridged ladder polymer **24** containing naphthalene diimide (NDI) building blocks, synthesized using the same method as **24** (Scheme 10).^17^ The β-branched 2-octyldodecyl alkyl side chain on the NDI nitrogens provided sufficient solubility for ladder polymer **25** in common organic solvents. It is worthy to note that the SEC-measured molecular weight of **25** (*M*_n_ = 14 kg mol^–1^) was overestimated due to the increased hydrodynamic radius of the rigid, ribbon-like backbone, giving a higher *M*_n_ than the precursor conjugated polymer (7.2 kg mol^–1^).

**Scheme 11:**
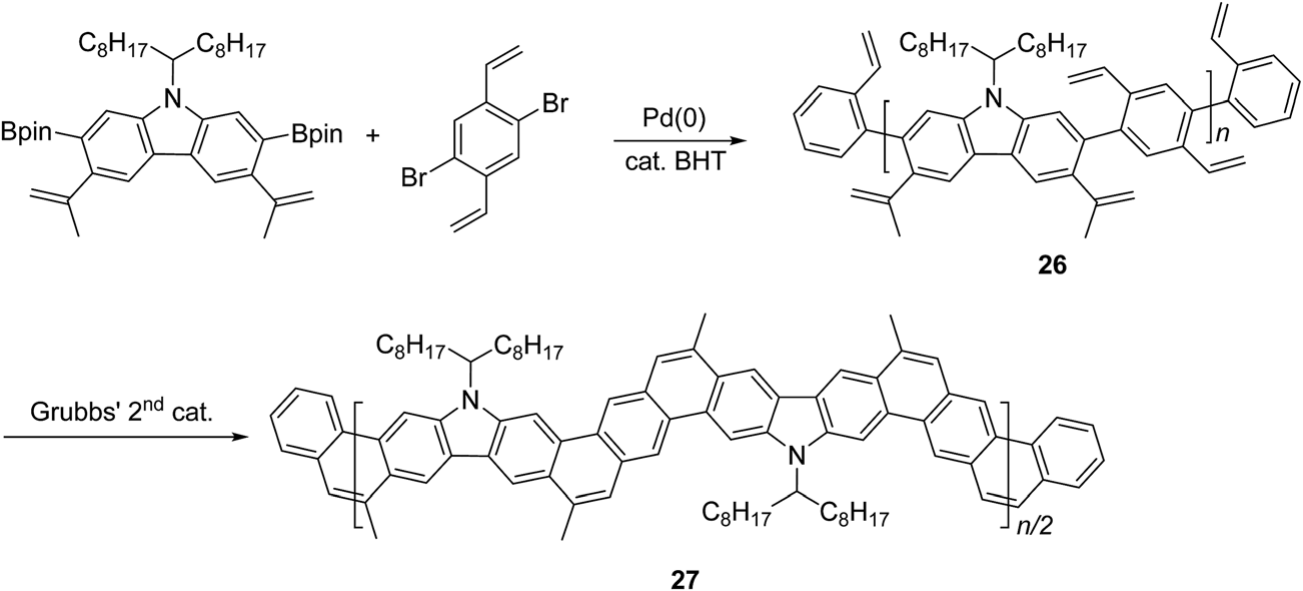
Synthesis of carbazole-based ladder polymer **27** by thermodynamically controlled ring-closing olefin metathesis.

Another method that has recently been recognized as an efficient strategy to construct fused-ring aromatic systems is the thermodynamically controlled ring-closing olefin metathesis (RCM) method.^54^ The dynamic nature of the RCM reaction can avoid the formation of cross-metathesis side-products and drives the reaction equilibrium to the desired fused-ring product, which sits in a deep energy sink because of additional aromaticity. Fang and coworkers reported the synthesis of carbazole-derived ladder polymer **27** by RCM from vinyl pendant precursor polymer **26** (Scheme 11).^7^ The single-stranded conjugated polymer **26** was prepared by Suzuki polymerization and endcapped using styrene derivatives. The reaction was conducted in the presence of a catalytic amount of butylated hydroxytoluene (BHT) as a radical scavenger, to avoid radical crosslinking of the styrene-like derivatives. Because of the strong solubilizing effect of the α-branched 1-octylnonyl group on carbazole, cLP **27** showed good solubility in common organic solvents at room temperature, allowing for solution analysis and processing. ^13^C NMR analysis of ^13^C isotope-enriched **27** revealed that the average number of possible unreacted vinyl groups in a single polymer chain was less than one (defect < 1% and DP^SEC^ = 23–27). Due to the minimum levels of unreacted defects, the polymer conformation was maintained in solution and the solid state giving nearly identical UV-vis spectra in both states with Stokes shift of only 1 nm.

Overall, the development of both single-step ladderization and stepwise polymerization followed by ladderization methods has steadily progressed in the past decade. Although the single-step approach is limited in reaction scope, the strategy of using simultaneous dynamic bonds should provide promising advancement for cLP synthesis. For the stepwise approach, various synthetic methods have been explored to expand the selection of kinetic ring annulation. Furthermore, recent examples of thermodynamic ring annulation have widened the scope of the synthesis of well-defined cLPs. As synthetic methods to develop a wider range of cLPs have improved, similar progress has occurred in their analytical and characterization techniques. Though the challenges originating from cLPs' highly rigid structures have prompted the rise of many new techniques, continued innovation of more advanced approaches is still a potentially impactful opportunity.

## 3. Challenges and issues

### 3-1. Structural defects

In conventional conjugated polymers, the planar aromatic repeating units tend to adopt non-zero dihedral angles between each building block due to torsional strain and thermodynamic fluctuation.^55^ Such torsional defects partially break the coherent π-conjugation of the backbone, shorten the effective conjugated lengths along the polymer chain, and decrease carrier mobilities.^8,56^ The torsional defects also perturb the intermolecular packing of the polymer materials, resulting in a higher energy barrier for the charge carriers and excitons to transport throughout the bulk material.^11,57^ These combined factors cause a much lower electronic performance of polymers compared to the theoretical value of a conjugated polymer chain.^9^ Unlike conventional conjugated polymers, ideal conjugated ladder polymers are torsional defect-free, maximizing π-electron delocalization. As a result, cLPs with a perfect structure should show increased electronic performance over conventional conjugated polymers. Such a defect-free cLP, however, is challenging to synthesize and characterize, as described above. Most of the reported cLPs are likely decorated with structural defects resulting from unreacted sites or side-reactions.

**Fig. 4:**
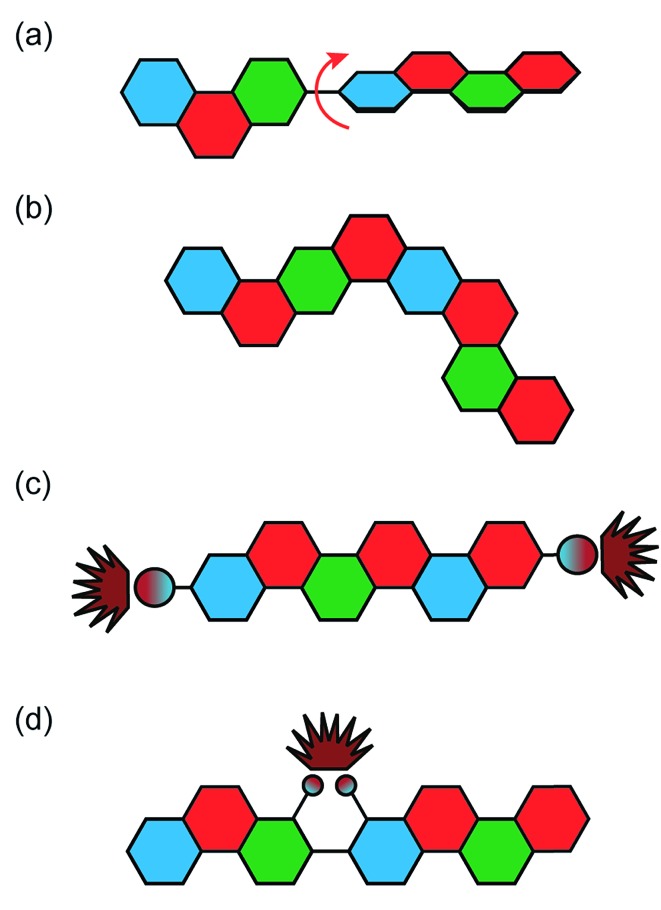
Defects common in cLPs. (a) Conjugation breaking torsional defects formed by incomplete ladderization or postsynthetic degradation, (b) regioisomeric structures created during nonregioselective syntheses, and non-conjugation breaking emissive defects (c) as a result of non-ladderized chain end groups, and (d) internally in the polymer chain.

Because of the significant impacts that polymer defects could impose on the material's properties, methods to remove or prevent these defects on cLPs are valuable. There are multiple ways in which defects can occur in a ladder polymer, including impurities in starting materials, incomplete or inefficient ladderization reactions, or a loss of solubility en route to the desired product (Fig. 4a and b). This leads to the demand of quantitative ladderization reactions, because even a small decrease in conversion from single-stranded conjugated polymer to cLP can cause multiple defects per strand if the molecular weight is high.

An alternative way to achieve low level of defect in cLP is to remove defect-containing macromolecules. In the case of cLP **27**, the small amount of polymer chains containing unreacted pendant vinyl groups was reacted in solution with azobisisobutyronitrile (AIBN) to initiate the free radical cross-linking of these vinyl groups. This reaction only takes place on the polymers containing vinyl defects, giving insoluble products that can be easily removed by filtration, leaving behind the pure defect free macromolecules in solution. In general, the defect removal process depends heavily on identifying reactions that selectively react with defect sites. For soluble cLPs, cross-linking the unwanted polymer chains through defect site reactions may be developed into a useful method for purification.

The end groups are also considered defects, because undesired end groups can affect the properties of the polymer by both acting as a charge trap as well as affecting long range packing in some conjugated polymers (Fig. 4c).^58–60^ In this context, end-capping a cLP during synthesis is sometimes necessary to lower the defect level for a better material performance.

In addition, some cLPs may develop defects after synthesis. The defect–property correlations have been investigated thoroughly in LPPP derived cLPs, by taking advantage of the large difference in emission characteristics between the pristine polymer and those with defects. For example, LPPPs **11** and **12** undergo oxidative degradation that can either break one of the strands of bonds and create a torsional defect or be oxidized into an emissive ketone defect. Both LPPPs **11** and **12** and alkylated polyfluorenes, which are photooxidized into fluorenones, have been studied to illustrate the effects of these emissive defects (Fig. 4d).^61^ After these oxidative defects are formed, both systems show a broad, low energy emission, sometimes called the green band. Though originally thought to be caused by excimers or other intermolecular interactions, more recent research has shown that it was solely the effect of ketonic defects acting as emissive traps.^62^ Such oxidation is much more likely to take place if the bridgehead carbon contains a hydrogen, as illustrated in HLPPP (R^3^ = H) **11**, which has a much stronger low-energy band than MeLPPP **12**. It is also possible for residual impurities to play a significant role in the degradation of cLPs. Ma's group showed that Pd(PPh_3_)_4_, a common aryl coupling catalyst, could trigger the oxidation of fluorene moieties into their fluorenone forms as well, demonstrating the critical role residual impurities could play in defect formation.^63^ PL lifetime measurements of defect formation were recently investigated by Lupton and coworkers using poly(9,9-dioctylfluorene) as a model system,^64^ showing evidence that the green band is not a single broad band but instead consists of multiple emitters, each at a discrete wavelength. Scherf and coworkers showed that these emissive properties are still maintained in the absence of any intermolecular interactions, further suggesting the lack of excimer involvement in emissions.^65^ To rectify this problem, the previously discussed spiro-LPPP **14** was synthesized through a slightly different, but defect-resistant synthetic scheme, and consequently showed a stable emission.^5,66^ These studies illustrated that defects can often impose significant impacts on the optical properties of cLPs.

### 3-2. Solubility and processing

The features of rigid backbones enhance the strong π–π interactions of cLPs and often result in their limited solubility caused by these strong intermolecular attractions.^1,67,68^ A notable example of a fully fused aromatic system is graphite, which is composed of π-stacked graphene layers and is apparently insoluble in any organic solvent. In addition, the presence of heteroaromatic repeating units may cause other interchain attractive interactions, *e.g*. hydrogen bonds and dipole–dipole interactions. Limited solubility of many cLPs due to these properties imposes challenges in processability and therefore in many practical applications.

A typical method to improve the solubility of cLPs is to introduce to the backbone flexible yet bulky side chains, which cause enough steric hindrance between chains to disrupt interchain aggregation.^17^ For example, in LPPPs **11** and **12**, the two alkyl groups installed on the quaternary sp^3^ carbon center can be viewed as a branched alkyl group. As the branching point is moved farther from the polymer chain, interchain π–π distance decreases, leading to a general trend of decreasing solubility but increasing charge carrier mobility in bulk.^69,70^ In addition, as alkyl chains grow longer, the amount of space taken up by nonconductive hydrocarbons increases, further separating conductive pathways. Therefore, a balance must be struck between solubility and device performance for a cLP that is designed for applications associated with electronic performances.^69,71–74^ In order to address this dilemma, side-chain engineering in terms of chemical structures, linkages to the backbone, and conformations need to be investigated extensively to best achieve the desired processability and properties of the cLP materials.

**Fig. 5:**
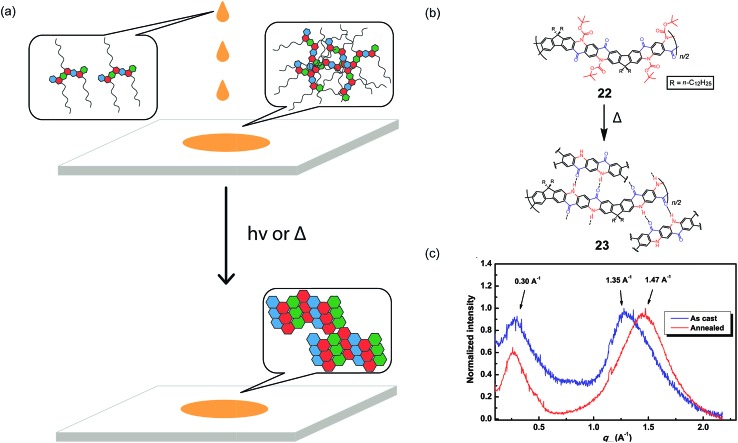
(a) Proposed illustration using cleavable side chains in cLPs processing to obtain a well-ordered, solvent resistant film from an easily processed material. (b) Schematic representation of Boc cleavage of **22** by thermal annealing in the solid state. (c) GIXD of the as-cast film of **22** (blue) in comparison with that of the annealed thin film (red). Reproduced from ref. 51 with permission from Elsevier.

A promising strategy to address the aforementioned challenge is the employment of cleavable side-chains. Cleavable solubilizing groups enable solution processability of the cLP materials and can also be easily removed after processing to potentially allow for efficient packing in the solid state (Fig. 5a). In addition, these polymers should possess significant solvent resistance after processing and side-chain cleavage,^75,76^ providing additional advantages for processing and operation in harsh conditions. In the example reported by Fang and coworkers, quinacridone derived ladder polymer **22** was rendered soluble by the incorporation of bulky Boc protecting groups that inhibit intermolecular hydrogen bonds (Fig. 5b).^51^ These Boc groups were then thermally cleaved to regenerate the hydrogen bonds and to produce thin films with remarkable solvent resistance. Grazing incidence X-ray diffraction (GIXD) measurements of the thermally annealed polymer thin films showed a decrease in the π–π stacking distance as a result of removal of the bulky Boc group (Fig. 5c).

### 3-3. Characterization of conformation and molecular weight

Because of the inherent rigid nature of cLPs, their conformations and dynamics in solution are expected to differ from prevailing non-rigid polymers significantly. Quantitatively, this difference should give a much higher Mark–Houwink exponent (0.8 < *a* < 2.0) for cLPs than that of flexible polymers (0.5 < *a* < 0.8). Correlation between hydrodynamic volumes and molecular weights for cLPs is therefore also drastically different from that of flexible polymers. As a result, traditional solution characterization techniques, such as SEC calibrated by polystyrene standards, cannot provide accurate depictions of the conformation and molecular weight for cLPs. Research has illustrated that when using multiple methods of analyzing molecular weights,^77–79^ the measured values vary between the different methods used. To date, a number of different methods have been applied to solve these issues, with varying levels of effectiveness.

**Fig. 6:**
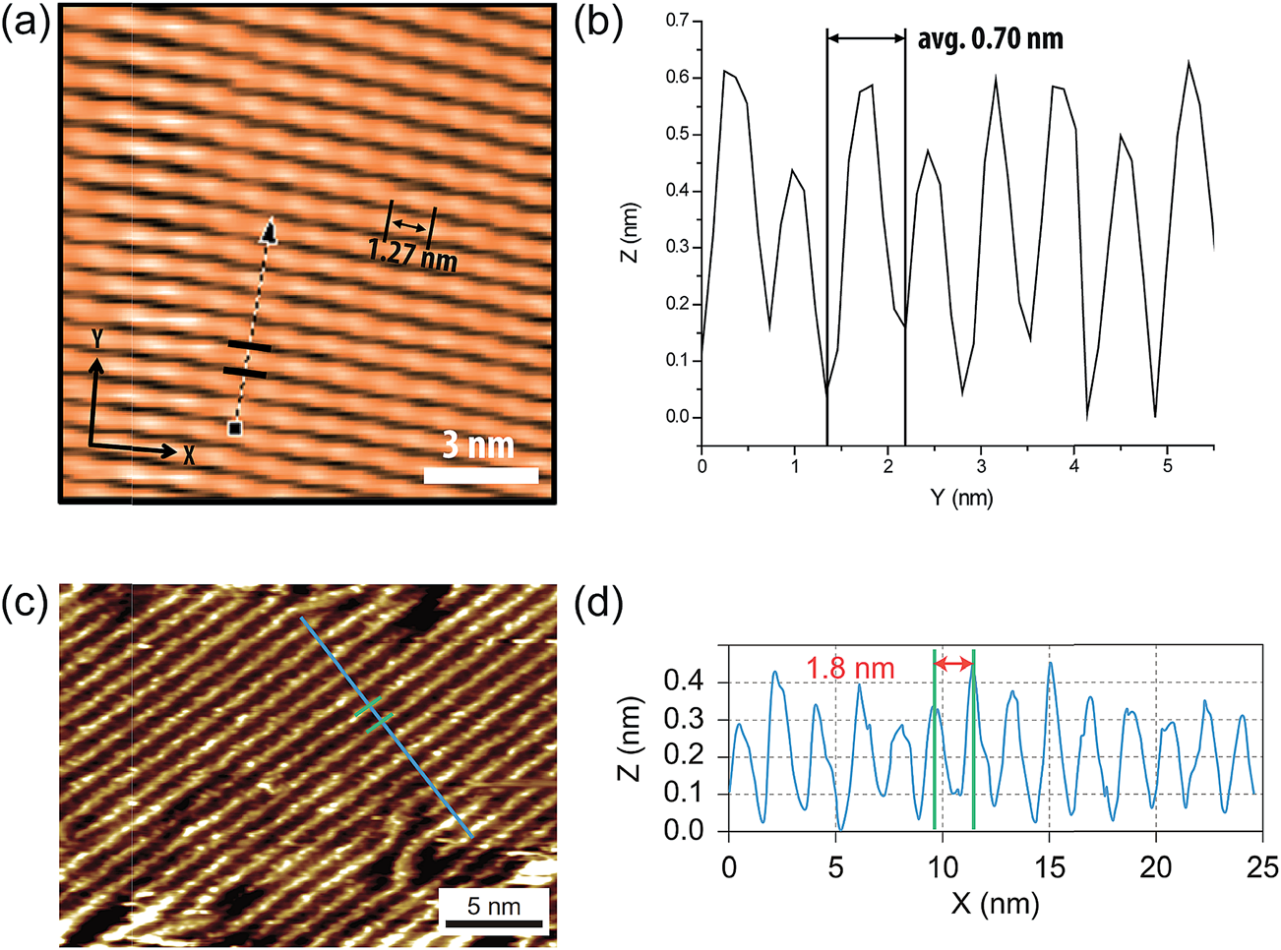
(a) STM images of cLP **27** on HOPG. (b) Section profile along the arrow line drawn in (a). (c) STM images of the graphene nanoribbon on HOPG from ref. 68. (d) Section profile along the blue line in (c). Reproduced from ref. 7 and 68 with permission from The Royal Society of Chemistry and Nature Publishing Group, respectively.

Small angle X-ray and neutron scattering (SAXS and SANS) have been applied to characterize the conformations and gain accurate molecular weights of rigid polymers. X-rays interact with the electron cloud of the molecule, while neutrons are scattered by elastic collisions with the nuclei of the material. The scattering intensity for various values of concentration (c) and scattering angle (*θ*) is plotted in a Zimm plot. Upon extrapolation to *c* = 0 and *θ* = 0, the intersection of the two lines is equal to 1/*M*_w_ and can therefore be used to determine accurate molecular weights. In addition, the slope of the *θ* = 0 line is proportional to the 2^nd^ virial coefficient. SANS in particular provides a unique method of characterization on partially deuterated sample in order to provide a higher contrast variation without largely changing the sample itself, due to the differences of neutron scattering between hydrogen and deuterium.^80^ Alternatively, a hydrogenated sample in deuterated solvent can also provide the needed contrast. These techniques were used, for example, to study the conformation of LPPP **12** in solution.^78^ The use of both methods showed a persistence length of approximately 6.5 nm, indicative of a 3D ribbon-like structure instead of a one-dimensional rigid rod. However, the complexity of the instrumentation and radiation sources necessary for these forms of measurement make it challenging to perform routine measurements on other cLP systems.

Simpler light scattering methods have also been utilized to characterize rigid polymers, including dynamic light scattering (DLS), which measures the anisotropic diffusion coefficients of the materials in solution and can be used to estimate the length of an idealized rigid rod.^81^ Because of the simplicity and accuracy of measurement, DLS has been used for other rod-like structures and represents an accessible method for the conformational analysis of cLPs.^82,83^

Other methods to characterize molecular weights of rigid ladder polymers include the use of osmometry or viscometry.^84,85^ These methods rely on the change in chemical potential of a solution of ladder polymers compared to a pure, non-theta solvent. Osmometry measures the osmotic pressure of a solution by change in volume through a semipermeable membrane (membrane osmometry) or of vapor pressure in a closed system (vapor phase osmometry). Viscometry measures the change in viscosity of varying concentrations of a polymer solution in order to find the polymer's intrinsic viscosity, and *via* the Mark–Houwink equation, the polymer's molecular weight. However, unreliable results may be obtained at either sufficiently high or low molecular weights depending on the method used. In addition, diffusion across the membrane of the osmometer can take extended time to reach an equilibrium.

In order to calculate the molecular weight and polydispersity index (PDI) of cLPs in a more rigorous manner, several advanced methods can be used. These include SEC with viscometer-assisted universal calibration^86,87^ or SEC coupled with multi-angle light scattering detectors.^88,89^ The employment of these methods for the characterization of cLPs, however, has not yet been well-established.

Structural elucidation has been another major challenge for the investigation of rigid cLPs. Both ^1^H and ^13^C NMR spectroscopy suffer from broad and low intensity signals, due largely to the limited solubility of the cLP samples and aggregation. This problem can be circumvented by the use of isotope labeling or alternative isotope measurements,^7^ in particular ^13^C or ^19^F, although the drawback for this method is sometimes tedious chemical synthesis. NMR analysis at higher temperature could also be employed to improve data quality by increasing the solubility and breaking up any aggregation. The incorporation of distinctive side chain or end-capping groups is another simple method to facilitate easier characterization. Alternatively, using the effects that rigid macromolecules have on NMR linewidths can also offer unique information of the polymers, such as the nature of solution aggregation or solid-state crystallinity.^90–92^

Surface probe microscopy can also be used to visualize the conformation of cLPs (Fig. 6). In particular, due to the conjugated and semiconducting nature of the backbones, individual polymer chains of some cLPs can be visualized by scanning tunneling microscopy (STM). STM analysis of cLP **27** on highly-ordered pyrolytic graphite (HOPG) showed the rigid and linear shape of the polymers with repeating units that aligned well with calculated dimensions of an oligomeric model.^7^ In addition, STM has found extended use in the visualization of graphene nanoribbons with improved resolution due to the extended conjugated system's favorable interaction with graphite substrates.^68,93^

In general, though many accurate techniques have been adopted for cLP characterization, the most common method remains the use of non-rigid SEC standards. Therefore, the widespread application of a universally accurate method would help standardize measurements across the breadth of the field, increasing the quality and efficiency of related research.

## 4. Applications of cLPs

As discussed in the previous sections, cLPs possess rigid and planar backbones with optimum π-electron delocalization and are free of torsional defects. From this perspective, cLPs are analogous to graphene nanoribbons, which combine the excellent charge transport property of graphene with opened band gaps as high-performance semiconducting materials.^94^ In addition, the rigid and planar backbones of cLPs also render extremely small Stokes shifts and high photoluminescence quantum yields.^95^ Furthermore, cLPs display potentially high thermal and optical stability as well as high resistance to chemical degradation.^2^ Such combination of unique properties of cLPs make them promising candidates for a wide range of applications.

### 4-1. Optical applications

Early examples have demonstrated that the superior optical properties of cLPs could lead to high performance in OLEDs.^2^ One area of focus has been the use of LPPPs as the active layer for OLEDs, taking advantage of the highly efficient yellow-green electroluminescence (EL).^14^ OLED devices fabricated using spiro-LPPP **14** as the active layer exhibited almost identical EL and PL spectra due to the minimal ketonic defects.^5^ This result indicated again the significant impact of defects on opto-electronic properties of cLPs and the importance of a low level of defects in these materials. Blue-green emitting derivative MeLPPP **12** showed EL efficiency up to 4%.^15^ To improve its processability, nanoparticles of MeLPPP **12** have also been prepared *via* miniemulsion and employed in an OLED device.^16^ Although the device showed a similar maximum brightness with that of the device fabricated from homogeneous solution, the turn-on voltage for the EL was reduced by 7 V. This improvement based on the nano-particulated ladder polymer was attributed to a better electron injection from the nanoparticles to the electrode.

**Fig. 7:**
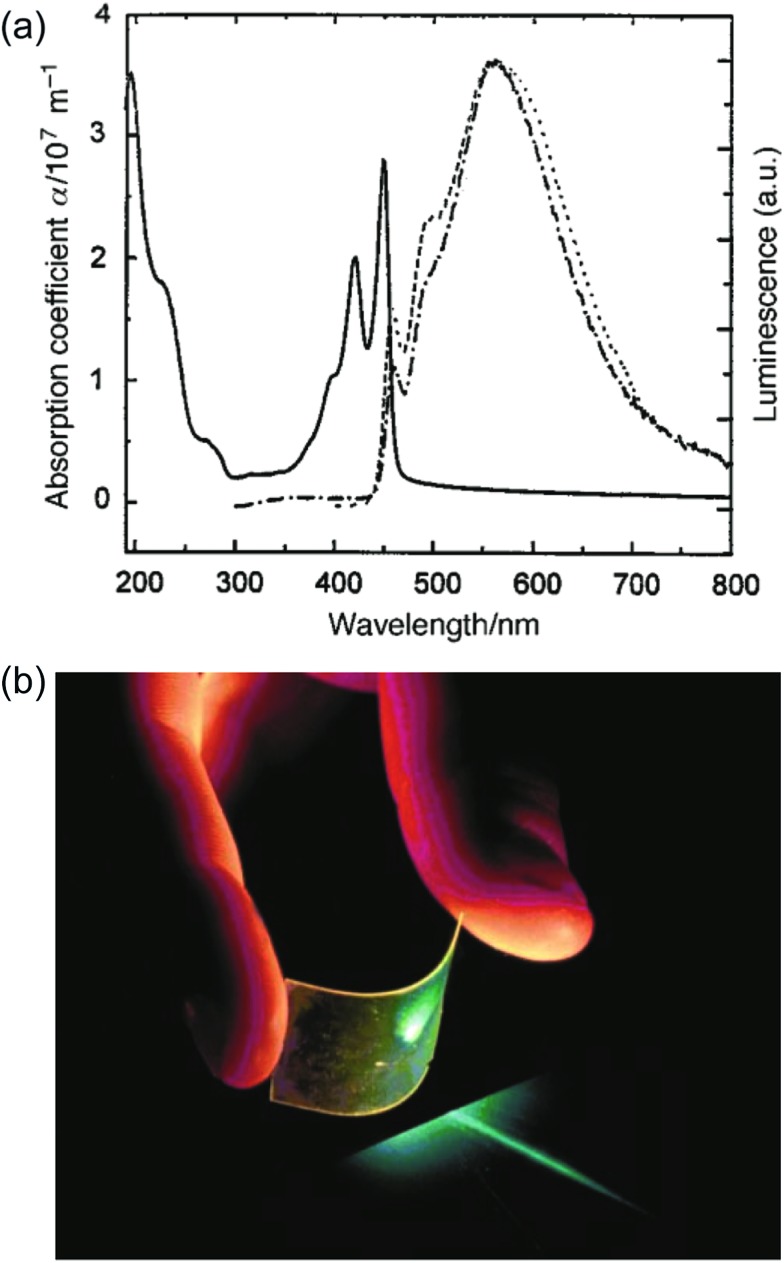
Optical properties of ladder polymers. (a) Absorption (—) and PL spectra ( <svg xmlns="http://www.w3.org/2000/svg" version="1.0" width="16.000000pt" height="16.000000pt" viewBox="0 0 16.000000 16.000000" preserveAspectRatio="xMidYMid meet"><metadata> Created by potrace 1.16, written by Peter Selinger 2001-2019 </metadata><g transform="translate(1.000000,15.000000) scale(0.005147,-0.005147)" fill="currentColor" stroke="none"><path d="M0 1440 l0 -80 360 0 360 0 0 80 0 80 -360 0 -360 0 0 -80z M1040 1440 l0 -80 360 0 360 0 0 80 0 80 -360 0 -360 0 0 -80z M2080 1440 l0 -80 320 0 320 0 0 80 0 80 -320 0 -320 0 0 -80z"/></g></svg> ) of a thin film of MeLPPP **12**. (b) A photo of a blue, flexible laser made from MeLPPP **12**. Reproduced from ref. 2 with permission from The Royal Society of Chemistry.

**Fig. 8:**
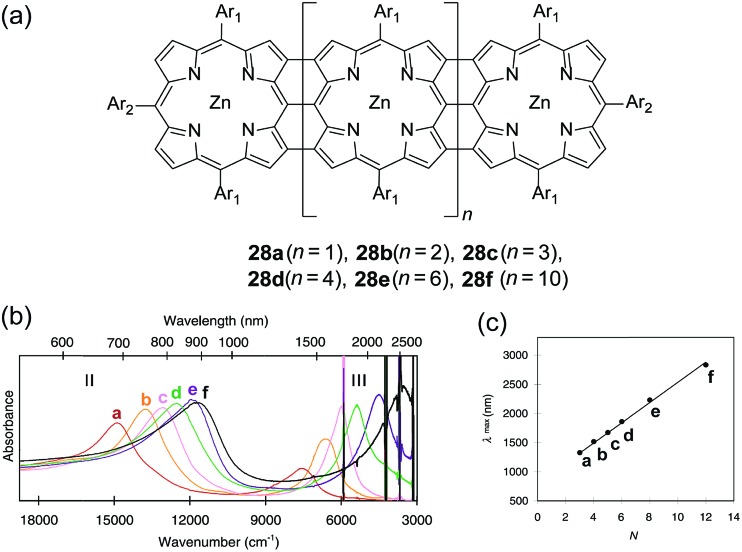
(a) Chemical structures of ladder-type porphyrins **28a–f**. (b) UV-vis-IR absorption spectra of porphyrins **28a–f**. (c) Plot of IR absorption maximum (band III) *versus* the number of porphyrins (*N*). Modified and reproduced from ref. 97 with permission from The American Association for the Advancement of Science.

Besides OLED applications, MeLPPP **12** has also been spin-cast onto a poly(ethylene terephthalate) substrate^96^ to afford a low-cost flexible distributed feedback laser. The laser emitted blue light centered at 487 nm with a linewidth of less than 0.4 nm (Fig. 7b). Another optically interesting example of cLPs is ladder-type triply fused porphyrin tapes (**28a–f**), which show a remarkably red-shifted absorption band in the IR region (Fig. 8).^97^ This IR absorption band was a result of the strong intramolecular electronic coupling and coplanar geometry of the molecule. The IR absorption maximum increases linearly with the number of porphyrin units, demonstrating the large increase in effective conjugation length. The porphyrin polymer **28f** with the highest number of repeating units showed an absorption peak around 3500 cm^–1^, making it a good candidate as an IR sensor. It is likely that **28f** has not yet reached the maximum effective conjugation length. Such a unique photophysical property of **28** was originated from the large coherent π-conjugation through triply fused backbone. As a result, the porphyrin tapes exhibited much faster internal conversion process and energy relaxation dynamics of the lowest excited states compared to that of monomer and the non-ladder type porphyrin oligomers.^98,99^

### 4-2. Electronic applications

cLPs show promise to reach a high charge carrier mobility compared to conventional conjugated polymers owing to a low level of torsional defects and a long effective conjugation length. Siebbeles and coworkers studied the intrachain mobility of LPPPs using time resolved microwave conductivity.^9^ With an average repeating unit of 54, the hole mobility along the MeLPPP **12** chain reached a remarkable value of 600 cm^2^ V^–1^ s^–1^. This high intrachain charge carrier mobility suggested that the bottleneck for the charge transport in bulk cLP materials is interchain charge transport.

**Fig. 9:**
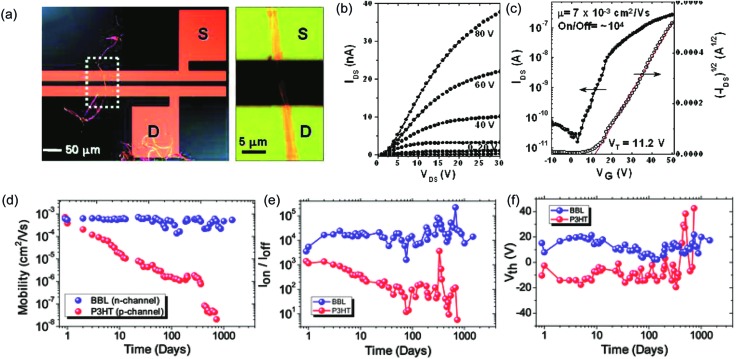
Performance of BBL **1** based OFETs. (a) A single BBL nanobelt bridging the source–drain electrode to generate a transistor. (b) Output curve of a typical BBL nanobelt transistor. (c) Transfer curve of the corresponding transistor. (d–f) Air stability analysis of P3HT and BBL transistors. Plot of (d) mobility, (e) current on/off ratio, and (f) threshold voltage as a function of time for both BBL and P3HT transistors. Reproduced from ref. 21 and 101 with permission from American Chemical Society and The Royal Society of Chemistry, respectively.

In terms of bulk electronic properties in OFETs, BBL **1** has been investigated extensively since the late 1980s.^100^ A series of improvement on charge transport properties were achieved by doping the BBL thin films with Lewis acid, but the overall mobility of fabricated devices was relatively low (10^–6^ to 10^–4^ cm^2^ V^–1^ s^–1^).^18,19^ In 2003, Jenekhe and coworkers achieved a record high electron mobility of BBL **1** up to 0.1 cm^2^ V^–1^ s^–1^ by doping and processing it with methanesulfonic acid (MSA).^20^ This value was 5 orders of magnitude higher than that obtained from a non-ladder type control polymer. Electron diffraction studies demonstrated a higher degree of crystallinity of the MSA-processed BBL film. This result also represented one of the highest electron transport mobilities achieved on an n-type polymer OFET at that time. Later on, Xia and Jenekhe and coworkers developed a high-yield solution-phase processing method to prepare BBL nanobelts with a good ambient stability.^21^ The BBL nanobelt was prepared by adding BBL in MSA solution dropwise to a CHCl_3_ and MeOH mixture with rapid stirring. These nanobelts can be suspended in water and used to fabricate OFET device *via* solution deposition. The n-type OFET device showed a mobility up to 7 × 10^–3^ cm^2^ V^–1^ s^–1^ and the on/off current ratios (*I*_on/off_) of 10^4^ (Fig. 9a–c). Moreover, in contrast with many n-type organic semiconductors which are sensitive to oxygen during operation,^101^ the BBL-based devices demonstrated exceptionally good stability in air for more than 4 years, even better than p-type polythiophene devices (Fig. 9d–f).^102^ The remarkable stability of BBL devices was attributed to its high degree of crystallinity and compact packing, which serves as a kinetic barrier to prevent oxygen from diffusing into the thin film.

Because of its excellent electronic properties and stability, BBL **1** has been used as a photoanode for the direct light-driven water oxidation reaction.^103^ The BBL photoanode showed good photoelectrochemical stability with no sign of degradation after 3 hours of water oxidation reaction. The electrical and thermoelectrical properties of a solution-processed BBL thin film was also studied very recently.^104^ After n-doping, the BBL thin film exhibited an electrical conductivity as high as 1.7 ± 0.6 S^–1^, three orders higher than that of NDI-based conventional conjugated polymer P(NDI2OD-T2). Density functional theory (DFT) calculation indicated that BBL possesses a much more delocalized polaron along its backbone than P(NDI2OD-T2), due to its coplanar backbone and low level of torsional defects. After optimizing, the thermoelectric power factor (*S* ^2^ *σ*) of BBL reached 0.43 μW m^–1^ K^–2^, much higher than those observed for non-ladder P(NDI2OD-T2).

Luscombe and coworkers reported the fabrication of an n-type OFET using an NDI-based ladder polymer **25**.^17^ OFET device fabrication using **25** from chlorobenzene solution by spin-coating exhibited an average electron mobility of 0.0026 cm^2^ V^–1^ s^–1^, which is three orders of magnitude larger than its non-ladder counterparts. The on/off current ratio of the device (*I*_on/off_ = 10^4^) was 2 orders of magnitude higher than that of the non-ladder type polymer.

**Fig. 10:**
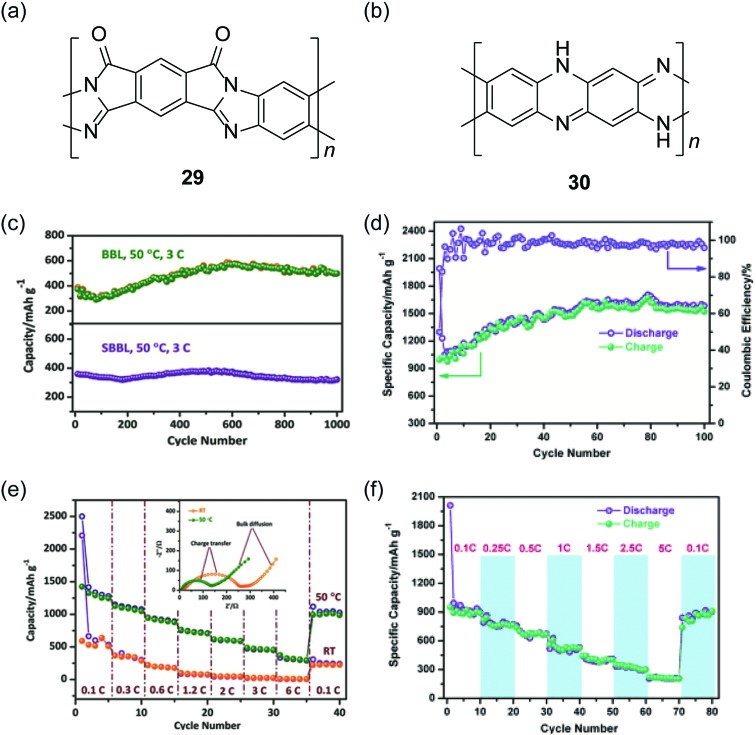
Chemical structures of (a) SBBL **29** and (b) PQL **30**. Electrochemical performance of ladder polymer nanoparticles. Cycling performance of (c) BBL **1**, SBBL **29** and (d) PQL **30** nanoparticles showing their superb stability. Rate capability of (e) BBL **1**, SBBL **29** and (f) PQL **30** nanoparticles. Reproduced from ref. 107 and 108 with permission from John Wiley and Sons.

Recently, cLPs with a large number of heteroatoms were found to be promising candidates for anode materials of lithium ion batteries. The prevailing electrodes for lithium ion batteries in the current market are graphite and LiCoO_2_. The theoretical capacities of these materials, however, are only about 372 mA h g^–1^ and 140 mA h g^–1^, respectively.^105^ One important strategy to increase the performance is to incorporate redox active heteroatom sites into fused-ring sp^2^ systems, such as nitrogen or sulfur. Fan and coworkers synthesized a polysulfur-grafted ladder poly(pyridinopyridine) by heating the precursor poly(acrylonitrile) (PAN) with sulfur at 350 °C.^106^ Although the constitutional structure of this ladder polymer is not well-defined, the high percentage of nitrogen and sulfur atoms permits multi-electron states for this polymer to give a high reversible capacity of 1750 mA h g^–1^. Yan and Zhang and coworkers exploited nanoparticles of BBL **1** and its derivative SBBL **29** as the anode materials of a rechargeable lithium ion battery.^107^ These ladder polymers showed a high capacity (1787 mA h g^–1^, 0.05C), a good charge rate (317 mA h g^–1^, 6C) and an excellent reversibility (1000 cycles, 496 mA h g^–1^) (Fig. 10c and e). Inspired by these results, PQL **30** was synthesized to represent a ladder polymer with a large number of nitrogen heteroatoms to serve as the lithium ion insertion sites.^108^ At the charge rate of 0.05C under 50 °C, the lithium half-cell made from PQL **30** nanoparticles exhibited a capacity of 1770 mA h g^–1^ (Fig. 10d). Moreover, PQL **30** maintained a reversible capacity (above 300 mA h g^–1^) at a high charge rate of 5C (Fig. 10f). The excellent electrochemical performances of cLP materials promise their applications as alternative electrodes for lithium ion batteries.

### 4-3. Outlook for potential applications

Despite the aforementioned examples, large-scale applications of cLPs have not been widely established yet, likely due to the synthetic and processing challenges. It is anticipated, however, that these issues can be solved using various chemical and engineering tools. Therefore, advancement along this direction together with the promising properties of cLPs could open up a number of intriguing opportunities for cLP applications.

First of all, cLPs can be viewed as functionalized fragments of single-wall carbon nanotubes (SWCNTs) or graphene nanoribbons. The exceptionally high Young's modulus (>1 TPa) of SWCNTs and graphene^109,110^ suggest that the simplest linear cLPs, polyacenes, could potentially possess good mechanical properties. DFT calculations on a polyacene resulted in a chain moduli as high as 745 GPa, close to that of (5,5) SWCNT (1046 GPa).^6^ Although polyacenes cannot be synthesized so far due to synthetic challenges and chemical stability issues,^111^ cLPs similar to polyacenes can be potentially prepared and should afford comparable mechanical performances.

Furthermore, cLPs are also promising candidates as precursors for high performance carbon fibers. Currently, most high-performance carbon fibers are produced from PAN.^112^ Before carbonization, PAN fibers undergo a stabilization process in which they are oxidized to form a ladder polymer-like structure.^113^ The progress of the ladderization reaction largely determines the quality of the carbon fiber. Therefore, carbonization of a defect-free ladder polymer with a well-controlled structure could potentially further enhance the mechanical properties of carbon fibers and enrich their functionalities.

Another potential application for cLPs is for organic photovoltaics (OPVs). Step-ladder polymers have been extensively studied for active layer materials in OPVs.^114^ For step-ladder polymers, the increase of the conjugation length of the polymer would effectively enhance charge separation and carrier mobility, contributing to a better power conversation efficiency of the OPVs.^115^ Compared to step-ladder polymers, cLPs possess an even more planarized structure, rendering a higher electron delocalization, larger charge carrier mobility, and better absorptivity, resulting in a potentially higher photovoltaic efficiency. OPV performances should become optimized as the active polymers approach a fully defect-free conjugated structure. In addition, the promising thermal and photo-stability of cLPs could serve as the key to address the stability issues of OPVs. Of course, potential morphological problems still remain when utilizing these highly rigid polymers in a device setting.

Furthermore, the rigidity and concomitant low disorder of cLPs also make it an ideal platform to study Bose–Einstein condensate (BEC) physics of exciton-polaritons. BECs of exciton-polaritons were normally observed in crystalline materials considering the high level of disorder in the condensed phase. Due to the complexity of crystal growth in the microcavity, investigating BEC physics was challenging. Stoferle and coworkers demonstrated exciton-polaritons' BEC state can be generated in an amorphous MeLPPP **12**, at room temperature, when coupling the polymer thin film to the confined photon mode of a Fabry–Perot microcavity.^116^ This study suggests cLPs can provide a new approach to study BEC physics with a simplified experimental condition.

## 5. Conclusions and outlook

This article summarizes important historical syntheses, examples of processing and applications of cLPs, as well as significant advances in the past decade. The intriguing properties of cLPs promise their future as next generation functional polymer materials. However, in order to maximize the potential of this class of materials, the challenges associated with the synthesis, structural defects, characterization, solubility, and processability of cLPs need to be addressed.

For the synthesis of a well-defined cLP, the development of synthetic strategies in either single-step ladderization or post-polymerization ladderization has seen significant progress. Although the single-step approach is limited by the availability of reactions suitable for the formation of multiple strands of bonds, the idea of using spontaneous intramolecular dynamic bonds enables the possibility of constructing a coplanar ladder-like structure through a single-step polymerization. The stepwise strategy – *i.e.*, polymerization followed by ladderization, – now could afford feasible approach to well-defined cLPs with fewer structural defects. More recently, thermodynamic ring annulation, including RCM, has widened the scope of well-defined cLP synthesis and promises the production of defect-free cLPs.

In order to analyze cLPs' highly rigid structures, analytical and characterization techniques have to evolve alongside the development of synthetic strategies. In contrast with conventional conjugated polymers, the conformation and dynamics of cLPs in solution are significantly different. Therefore, common characterization methods such as SEC with polystyrene standards do not provide accurate depictions of the conformation and molecular weight for cLPs. Several different analytical methods have been applied to overcome the limited effectiveness of SEC. However, the complexity of the instrumentation restricts extensive routine measurements for cLPs. The visualization of rigid ribbon-like structures was also studied by STM. Even though STM displayed detailed conformations of rigid structures at a small scale on solid substrate, it cannot provide an all-encompassing analysis of the entire batch or that in solution. ^1^H NMR spectroscopy provides rich characterization information of cLPs, but it is often limited by the line broadening and low signal/noise ratio. Hence, the development of accurate characterization methods for cLP characterization is as important as the development of synthetic strategies.

Owing to the limited solubility of cLPs in common organic solvents, only a few successful processing methods were reported before LPPPs were first reported. Because LPPPs are usually soluble in common organic solvents, their electronic and optical properties have been extensively studied. Particularly, MeLPPP **12** has been used for many applications such as OLEDs and OFETs and showed excellent optoelectronic properties. Moreover, the advances made in processing methods allowed processability of insoluble cLPs like BBL **1** through the formation of nanoparticles, leading to devices showing exceptional air stability for multiple years. More recently, cLPs have been applied as electrodes for lithium ion batteries and demonstrated superior capacity and reversibility. Despite these exciting progresses, the applications of cLPs have not been fully explored yet due to previously mentioned challenges.

Overall, cLPs possess great potential in applications at multiple fronts due to their promising superior properties with remarkable stability. Throughout 50 years of exploration and development, we have seen the promising aspects of cLPs as functional organic materials. We believe, however, that many more thrilling discoveries in this class of materials will be made in the future.

## References

[cit1] Yu L., Chen M., Dalton L. R. (1990). Chem. Mater..

[cit2] Scherf U. (1999). J. Mater. Chem..

[cit3] Grimsdale A. C., Müllen K. (2007). Macromol. Rapid Commun..

[cit4] Jones R. S. R. G., Wilks E. S., Hess M., Kitayama T. and Metanomski W. V., Compedium of polymer terminology and nomenclature IUPAC recommendations 2008, The Royal Society of Chemistry, Cambridge, UK, 2008.

[cit5] Wu Y., Zhang J., Fei Z., Bo Z. (2008). J. Am. Chem. Soc..

[cit6] Zeng S. Z., Jin N. Z., Zhang H. L., Hai B., Chen X. H., Shi J. L. (2014). RSC Adv..

[cit7] Lee J., Rajeeva B. B., Yuan T., Guo Z.-H., Lin Y.-H., Al-Hashimi M., Zheng Y., Fang L. (2016). Chem. Sci..

[cit8] Grozema F. C., van Duijnen P. T., Berlin Y. A., Ratner M. A., Siebbeles L. D. A. (2002). J. Phys. Chem. B.

[cit9] Prins P., Grozema F. C., Schins J. M., Patil S., Scherf U., Siebbeles L. D. A. (2006). Phys. Rev. Lett..

[cit10] Samiullah M., Moghe D., Scherf U., Guha S. (2010). Phys. Rev. B: Condens. Matter.

[cit11] Bjorgaard J. A., Köse M. E. (2013). J. Phys. Chem. A.

[cit12] Van Deusen R. L. (1966). J. Polym. Sci., Part B: Polym. Lett..

[cit13] Schlüter A. D. (1991). Adv. Mater..

[cit14] Grem G., Leising G. (1993). Synth. Met..

[cit15] Leising G., Tasch S., Meghdadi F., Athouel L., Froyer G., Scherf U. (1996). Synth. Met..

[cit16] Piok T., Gamerith S., Gadermaier C., Plank H., Wenzl F. P., Patil S., Montenegro R., Kietzke T., Neher D., Scherf U., Landfester K., List E. J. W. (2003). Adv. Mater..

[cit17] Durban M. M., Kazarinoff P. D., Segawa Y., Luscombe C. K. (2011). Macromolecules.

[cit18] Babel A., Jenekhe S. A. (2002). Adv. Mater..

[cit19] Chen X. L., Bao Z., Schön J. H., Lovinger A. J., Lin Y.-Y., Crone B., Dodabalapur A., Batlogg B. (2001). Appl. Phys. Lett..

[cit20] Babel A., Jenekhe S. A. (2003). J. Am. Chem. Soc..

[cit21] Briseno A. L., Mannsfeld S. C. B., Shamberger P. J., Ohuchi F. S., Bao Z., Jenekhe S. A., Xia Y. (2008). Chem. Mater..

[cit22] Stille J. K., Mainen E. L. (1966). J. Polym. Sci., Part B: Polym. Lett..

[cit23] Stille J. K., Mainen E. L. (1966). J. Polym. Sci., Part B: Polym. Lett..

[cit24] Kim O.-K. (1985). J. Polym. Sci., Polym. Lett. Ed..

[cit25] Schlüter A. D., Loffler M., Enkelmann V. (1994). Nature.

[cit26] Schlicke B., Schirmer H., Schlüter A. D. (1995). Adv. Mater..

[cit27] Delnoye D. A. P., Sijbesma R. P., Vekemans J. A. J. M., Meijer E. W. (1996). J. Am. Chem. Soc..

[cit28] Pieterse K., Vekemans J. A. J. M., Kooijman H., Spek A. L., Meijer E. W. (2000). Chem.–Eur. J..

[cit29] Vetrichelvan M., Valiyaveettil S. (2005). Chem.–Eur. J..

[cit30] Wakamiya A., Taniguchi T., Yamaguchi S. (2006). Angew. Chem., Int. Ed..

[cit31] Tian Y.-H., Kertesz M. (2009). Macromolecules.

[cit32] Zhu C., Guo Z.-H., Mu A. U., Liu Y., Wheeler S. E., Fang L. (2016). J. Org. Chem..

[cit33] Crossley D. L., Cade I. A., Clark E. R., Escande A., Humphries M. J., King S. M., Vitorica-Yrezabal I., Ingleson M. J., Turner M. L. (2015). Chem. Sci..

[cit34] Scherf U., Müllen K. (1991). Makromol. Chem., Rapid Commun..

[cit35] Yuan Z., Xiao Y., Yang Y., Xiong T. (2011). Macromolecules.

[cit36] Kass K. J., Forster M., Scherf U. (2016). Angew. Chem., Int. Ed..

[cit37] Nehls B. S., Füldner S., Preis E., Farrell T., Scherf U. (2005). Macromolecules.

[cit38] Patil S. A., Scherf U., Kadashchuk A. (2003). Adv. Funct. Mater..

[cit39] Forster M., Annan K. O., Scherf U. (1999). Macromolecules.

[cit40] Fiesel R., Huber J., Scherf U. (1996). Angew. Chem., Int. Ed..

[cit41] Scherf U., Müllen K. (1992). Polymer.

[cit42] Cho S. Y., Grimsdale A. C., Jones D. J., Watkins S. E., Holmes A. B. (2007). J. Am. Chem. Soc..

[cit43] List E. J. W., Guentner R., Freitas P. S. d., Scherf U. (2002). Adv. Mater..

[cit44] Scherf U., List E. J. W. (2002). Adv. Mater..

[cit45] Qiu S., Lu P., Liu X., Shen F., Liu L., Ma Y., Shen J. (2003). Macromolecules.

[cit46] Chen Y., Huang W., Li C., Bo Z. (2010). Macromolecules.

[cit47] Chmil K., Scherf U. (1993). Makromol. Chem., Rapid Commun..

[cit48] Chmil K., Scherf U. (1997). Acta Polym..

[cit49] Goldfinger M. B., Swager T. M. (1994). J. Am. Chem. Soc..

[cit50] Debad J. D., Bard A. J. (1998). J. Am. Chem. Soc..

[cit51] Zou Y., Ji X., Cai J., Yuan T., Stanton D. J., Lin Y.-H., Naraghi M., Fang L. (2017). Chem.

[cit52] Rowan S. J., Cantrill S. J., Cousins G. R. L., Sanders J. K. M., Stoddart J. F. (2002). Angew. Chem., Int. Ed..

[cit53] Tour J. M., Lamba J. J. S. (1993). J. Am. Chem. Soc..

[cit54] Bonifacio M. C., Robertson C. R., Jung J.-Y., King B. T. (2005). J. Org. Chem..

[cit55] Tsai F. C., Chang C. C., Liu C. L., Chen W. C., Jenekhe S. A. (2005). Macromolecules.

[cit56] Streifel B. C., Peart P. A., Martínez Hardigree J. F., Katz H. E., Tovar J. D. (2012). Macromolecules.

[cit57] Wood S., Kim J. H., Hwang D. H., Kim J. S. (2015). Chem. Mater..

[cit58] Wang Q., Zhang B., Liu L., Chen Y., Qu Y., Zhang X., Yang J., Xie Z., Geng Y., Wang L., Wang F. (2012). J. Phys. Chem. C.

[cit59] Asaoka S., Takeda N., Iyoda T., Cook A. R., Miller J. R. (2008). J. Am. Chem. Soc..

[cit60] Koldemir U., Puniredd S. R., Wagner M., Tongay S., McCarley T. D., Kamenov G. D., Müllen K., Pisula W., Reynolds J. R. (2015). Macromolecules.

[cit61] Grisorio R., Piliego C., Striccoli M., Cosma P., Fini P., Gigli G., Mastrorilli P., Suranna G. P., Nobile C. F. (2008). J. Phys. Chem. C.

[cit62] Lupton J. M. (2002). Chem. Phys. Lett..

[cit63] Liu L., Yang B., Zhang H., Tang S., Xie Z., Wang H., Wang Z., Lu P., Ma Y. (2008). J. Phys. Chem. C.

[cit64] Adachi T., Vogelsang J., Lupton J. M. (2014). J. Phys. Chem. Lett..

[cit65] Becker K., Lupton J. M., Feldmann J., Nehls B. S., Galbrecht F., Gao D. Q., Scherf U. (2006). Adv. Funct. Mater..

[cit66] Wang B., Forster M., Preis E., Wang H., Ma Y., Scherf U. (2009). J. Polym. Sci., Part A: Polym. Chem..

[cit67] Simpson C. D., Brand J. D., Berresheim A. J., Przybilla L., Rader H. J., Müllen K. (2002). Chem.–Eur. J..

[cit68] Narita A., Feng X., Hernandez Y., Jensen S. A., Bonn M., Yang H., Verzhbitskiy I. A., Casiraghi C., Hansen M. R., Koch A. H. R., Fytas G., Ivasenko O., Li B., Mali K. S., Balandina T., Mahesh S., De Feyter S., Müllen K. (2014). Nat. Chem..

[cit69] Kang I., Yun H.-J., Chung D. S., Kwon S.-K., Kim Y.-H. (2013). J. Am. Chem. Soc..

[cit70] Liu L., Han T., Wu X., Qiu S., Wang B., Hanif M., Xie Z., Ma Y. (2015). J. Phys. Chem. C.

[cit71] Lee J., Han A. R., Yu H., Shin T. J., Yang C., Oh J. H. (2013). J. Am. Chem. Soc..

[cit72] Lei T., Dou J.-H., Pei J. (2012). Adv. Mater..

[cit73] Lei T., Wang J. Y., Pei J. (2014). Acc. Chem. Res..

[cit74] Mei J., Bao Z. (2013). Chem. Mater..

[cit75] Guo Z.-H., Ai N., McBroom C. R., Yuan T., Lin Y.-H., Roders M., Zhu C., Ayzner A. L., Pei J., Fang L. (2016). Polym. Chem..

[cit76] Smith Z. C., Meyer D. M., Simon M. G., Staii C., Shukla D., Thomas S. W. (2015). Macromolecules.

[cit77] Godt A., Schlüter A.-D. (1992). Makromol. Chem..

[cit78] Hickl P., Ballauff M., Scherf U., Müllen K., Lindner P. (1997). Macromolecules.

[cit79] Wong M., Hollinger J., Kozycz L. M., McCormick T. M., Lu Y., Burns D. C., Seferos D. S. (2012). ACS Macro Lett..

[cit80] März K., Lindner P., Urban G., Ballauff M., Kugler J., Fischer E. W. (1993). Acta Polym..

[cit81] Liu T., Xiao Z. (2012). Macromol. Chem. Phys..

[cit82] Trachtenberg S. and Hammel I., in Microscopy: Science, Technology, Applications and Education, ed. A. D. Méndez-Vilas and J. Díaz, 2010, vol. 3, pp. 1690–1695.

[cit83] Shetty A. M., Wilkins G. M. H., Nanda J., Solomon M. J. (2009). J. Phys. Chem. C.

[cit84] Su W.-F., in Principles of Polymer Design and Synthesis, Springer Berlin Heidelberg, Berlin, Heidelberg, 2013, vol. 2, pp. 9–26, 10.1007/978-3-642-38730-2.

[cit85] Ebdon J. R. (1992). Polym. Int..

[cit86] Striegel A. M. (2016). Chromatographia.

[cit87] Vanhee S., Rulkens R., Lehmann U., Rosenauer C., Schulze M., Köhler W., Wegner G. (1996). Macromolecules.

[cit88] Molina R., Gómez-Ruiz S., Montilla F., Salinas-Castillo A., Fernández-Arroyo S., Ramos M. d. M., Micol V., Mallavia R. (2009). Macromolecules.

[cit89] Cotts P. M., Swager T. M., Zhou Q. (1996). Macromolecules.

[cit90] Gao B., Wang M., Cheng Y., Wang L., Jing X., Wang F. (2008). J. Am. Chem. Soc..

[cit91] Collison C. J., Rothberg L. J., Treemaneekarn V., Li Y. (2001). Macromolecules.

[cit92] Dudenko D., Kiersnowski A., Shu J., Pisula W., Sebastiani D., Spiess H. W., Hansen M. R. (2012). Angew. Chem., Int. Ed..

[cit93] Chen L., Hernandez Y., Feng X., Müllen K. (2012). Angew. Chem., Int. Ed..

[cit94] Han M. Y., Özyilmaz B., Zhang Y., Kim P. (2007). Phys. Rev. Lett..

[cit95] Grimsdale A. C., Chan K. L., Martin R. E., Jokisz P. G., Holmes A. B. (2009). Chem. Rev..

[cit96] Kallinger C., Hilmer M., Haugeneder A., Perner M., Spirkl W., Lemmer U., Feldmann J., Scherf U., Müllen K., Gombert A., Wittwer V. (1998). Adv. Mater..

[cit97] Tsuda A., Osuka A. (2001). Science.

[cit98] Cho H. S., Jeong D. H., Cho S., Kim D., Matsuzaki Y., Tanaka K., Tsuda A., Osuka A. (2002). J. Am. Chem. Soc..

[cit99] Tanaka T., Osuka A. (2015). Chem. Soc. Rev..

[cit100] Jenekhe S. A., Tibbetts S. J. (1988). J. Polym. Sci., Part B: Polym. Phys..

[cit101] Bao Z. (2000). Adv. Mater..

[cit102] Briseno A. L., Kim F. S., Babel A., Xia Y. N., Jenekhe S. A. (2011). J. Mater. Chem..

[cit103] Bornoz P., Prévot M. S., Yu X., Guijarro N., Sivula K. (2015). J. Am. Chem. Soc..

[cit104] Wang S., Sun H., Ail U., Vagin M., Persson P. O., Andreasen J. W., Thiel W., Berggren M., Crispin X., Fazzi D., Fabiano S. (2016). Adv. Mater..

[cit105] Xie J., Zhao C.-E., Lin Z.-Q., Gu P.-Y., Zhang Q. (2016). Chem.–Asian J..

[cit106] Wang L., He X., Sun W., Li J., Gao J., Tian G., Wang J., Fan S. (2013). RSC Adv..

[cit107] Wu J., Rui X., Wang C., Pei W.-B., Lau R., Yan Q., Zhang Q. (2015). Adv. Energy Mater..

[cit108] Wu J., Rui X., Long G., Chen W., Yan Q., Zhang Q. (2015). Angew. Chem., Int. Ed..

[cit109] Treacy M. M. J., Ebbesen T. W., Gibson J. M. (1996). Nature.

[cit110] Lee C., Wei X. D., Kysar J. W., Hone J. (2008). Science.

[cit111] Tonshoff C., Bettinger H. F. (2010). Angew. Chem., Int. Ed..

[cit112] Nataraj S. K., Yang K. S., Aminabhavi T. M. (2012). Prog. Polym. Sci..

[cit113] Dalton S., Heatley F., Budd P. M. (1999). Polymer.

[cit114] Wu J. S., Cheng S. W., Cheng Y. J., Hsu C. S. (2015). Chem. Soc. Rev..

[cit115] Li Y. X., Yao K., Yip H. L., Ding F. Z., Xu Y. X., Li X. S., Chen Y., Jen A. K. Y. (2014). Adv. Funct. Mater..

[cit116] Plumhof J. D., Stoferle T., Mai L., Scherf U., Mahrt R. F. (2014). Nat. Mater..

